# A Review of Optical Fibre Ethanol Sensors: Current State and Future Prospects

**DOI:** 10.3390/s22030950

**Published:** 2022-01-26

**Authors:** Sanober Farheen Memon, Ruoning Wang, Bob Strunz, Bhawani Shankar Chowdhry, J. Tony Pembroke, Elfed Lewis

**Affiliations:** 1 Optical Fibre Sensors Research Centre, University of Limerick, V94 T9PX Limerick, Ireland; ruonwang@hrbeu.edu.cn; 2 Department of Electronic and Computer Engineering, University of Limerick, V94 T9PX Limerick, Ireland; Bob.Strunz@ul.ie; 3 Key Laboratory of In-Fiber Integrated Optics of Ministry of Education, College of Physics and Optoelectronic Engineering, Harbin Engineering University, Harbin 150001, China; 4 NCRA-CMS Lab, IICT, Mehran University of Engineering and Technology, Jamshoro 76062, Sindh, Pakistan; bhawani.chowdhry@faculty.muet.edu.pk; 5 Department of Chemical Sciences and Bernal Institute, University of Limerick, V94 T9PX Limerick, Ireland; Tony.Pembroke@ul.ie

**Keywords:** ethanol sensing, optical fibre sensors, absorption-based sensors, interferometric sensors, fibre grating sensors, plasmonic sensors

## Abstract

A range of optical fibre-based sensors for the measurement of ethanol, primarily in aqueous solution, have been developed and are reviewed here. The sensing approaches can be classified into four groups according to the measurement techniques used, namely absorption (or absorbance), external interferometric, internal fibre grating and plasmonic sensing. The sensors within these groupings can be compared in terms of their characteristic performance indicators, which include sensitivity, resolution and measurement range. Here, particular attention is paid to the potential application areas of these sensors as ethanol production is globally viewed as an important industrial activity. Potential industrial applications are highlighted in the context of the emergence of the internet of things (IoT), which is driving widespread utilization of these sensors in the commercially significant industrial and medical sectors. The review concludes with a summary of the current status and future prospects of optical fibre ethanol sensors for industrial use.

## 1. Introduction

Ethanol is a colourless organic chemical, which is often referred to as alcohol or ethyl alcohol. Its attractive solvent properties of being easily soluble in water and other organic compounds means that it is one of the key chemicals used in modern industrial processes and consumer products. Its uses throughout society are manifold, including as a preservative, an anti-bacterial agent, an astringent in personal care products, as an antidote, an anti-infective and rubbing alcohol in medicines, as a solvent in paints, lacquers and varnishes, as well as an ingredient in intoxicating alcohol beverages and as an additive for flavouring and preserving food [1]. Since the 1970s, interest in the use of ethanol as a renewable fuel or partial substitute for gasoline has grown significantly. It is considered widely as a renewable alternative for fossil-based chemicals such as bioplastics and an additive for ethanol fuel blends [2,3]. In addition, the traditional drinks industry produces ethanol from yeast fermentation in brewing and wine manufacture and the distillation of spirits leads to a huge range of ethanol fortified alcoholic beverages [4].

The proliferation of ethanol uses as described above also increases the demand for its measurement in other sectors such as environmental health and safety, emission control, new biofuel production processes, in pharmaceutical and industrial product development, in breath analysis and in food quality assessment [5]. Analysis of ethanol in many processes is necessarily mandated by the relevant regulating agencies, e.g., the food and drugs administration (FDA) in the USA. A variety of methods can be used to measure the concentration of ethanol in aqueous solution. Commonly used detection methods include enzymatic measurement [6], Raman spectroscopy [7], UV/NIR spectroscopy [8], dichromatic oxidation spectrophotometry [9], refractive index (RI) analysis [8], gas chromatography (GC) [10], high-performance liquid chromatography (HPLC) [11], pycnometry [12], densimetry [12], hydrometry [12], capillary electrophoresis [13] and colorimetric methods [14]. However, the use of these methods has many disadvantages including low reproducibility, potential sample loss, long analysis time, complex offline sample preparation procedures and bulky and expensive instrumentation. Dichromatic oxidation spectrophotometry, pycnometry, hydrometry and densimetry suffer from large sample loss and require a moderate to long time for analysis [9,12,15,16]. The accuracy of the measurement is also highly dependent on the operator’s knowledge and the sample’s temperature [17]. Enzymatic methods are characteristically known for their low accuracy, reproducibility and enzyme stability. Modular Raman spectrometry necessitates precautionary measures for laser use and yield a detection limit of only 1% (*v*/*v*) ethanol [18,19]. Complicated calibration procedures are needed for near-infrared spectroscopy and, hence, it can be expensive and time consuming [20]. On the other hand, RI analysis is a relatively simple method, but the accuracy is highly dependent on temperature and can only be used for simple solvents (as opposed to complex mixtures) [21]. When compared to standard chromatography techniques such as GC and HPLC, capillary electrophoresis has lower accuracy. GC is currently considered the most reliable method for ethanol concentration measurement in clinical samples and for alcoholic drinks and is, therefore, the most widely used. Despite the benefits of chromatography techniques, they can be relatively slow (often requiring pre-concentration), complex and costly due to the large quantities of expensive organics needed [22]. Ethanol detection using stimuli-responsive hydrogels and piezoresistive pressure sensors has also been recently reported, where the ethanol concentration of a vodka product (“Wodka Gorbatschow”) with a specified value of 37.5 vol% ethanol was measured [23]. Many industries and research sectors, e.g., alcoholic beverage production and clinical/medical applications, require a simpler, more convenient and higher throughput determination of ethanol concentration [9]. The advantages and disadvantages of the commonly used ethanol detection methods are summarised in Table 1.

Recent advances in industrial practice including the emergence of Industry 4.0 for process automation and manufacture has meant that real-time monitoring is a crucial part of those processes. Big data is a major element of the Industrial Internet of Things (IIoT), which results from the generation and collection of massive data sets from sensors used in diagnosis, process monitoring, product manufacturing, health, safety, and quality control. Bulk optical sensing technology has the unique advantage of being immune to external electromagnetic interference and is potentially highly accurate, specifically in resolving very small changes in RI compared to existing commercially available technologies, but typically requires delicate alignment and coupling mechanisms, which increase sensor size, complexity and reduce stability, e.g., through susceptibility to mechanical vibration. On the other hand, optical sensors based on optical fibres are becoming a highly versatile, rugged and potentially cost-effective alternative due to their capability for being miniaturised, readily integrated with electro-optical components or electronic systems, feasible for real-time and remote sensing, and light weight, as well as having a minimised need for precise alignment and coupling [30,31].

**Figure 1:**
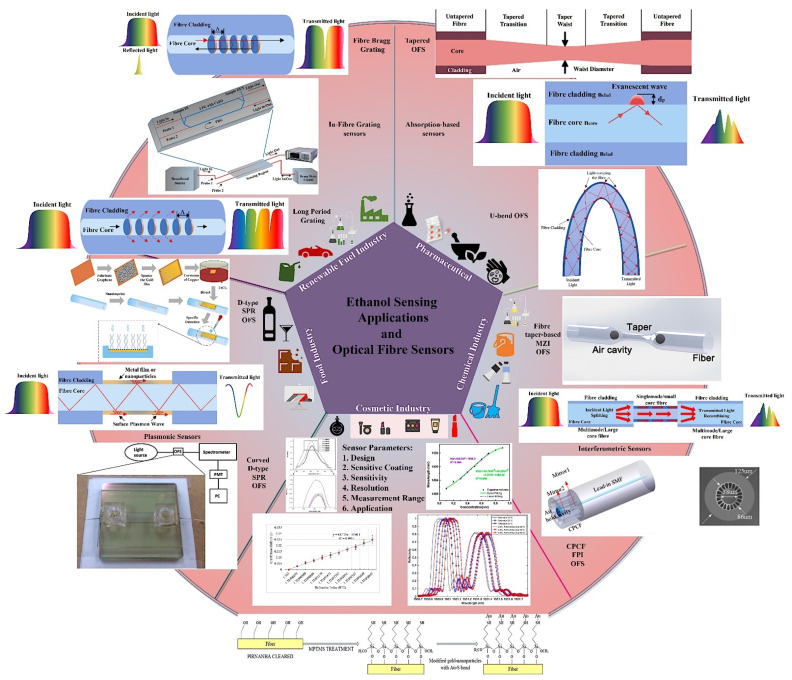
Optical fibre ethanol sensing schemes, applications of ethanol sensing and sensor parameters. Some data extracted from Refs. [35,36,37,38,39,40,41,42,43].

Optical fibres have been investigated for their potential use in sensing applications since the early 1980s, and several advances have been made in the fields of optical fibre chemical sensing and biosensing [32]. At that time, researchers also started exploring the concept of optical fibre-based ethanol sensors [33,34]. Wolfbies et al. (1988) used enzymatic oxidation of ethanol to create an optical fibre ethanol biosensor [34]. The sensor layer included an oxygen-sensitive fluorescing indicator that detected a drop in local oxygen partial pressure due to enzymatic oxidation. The sensor detected ethanol concentrations in the range of 50 to 500 mmol L^−1^, with an accuracy of ±4 mmol L^−1^ at 100 mmol L^−1^. Since then, a diverse range of mechanisms have been investigated for measuring ethanol concentration using optical fibre sensors and, in some cases, real applications have been further explored using these sensing schemes. In general, these sensors have delivered encouraging results and demonstrated great potential for ethanol sensing in a wide range of applications. Figure 1 is a graphical summary of a selection of applications for ethanol sensing and optical fibre sensing schemes utilised for ethanol sensing extracted from recent literature [35,36,37,38,39,40,41,42,43] and includes sensor design parameters and typical output responses. Several articles have recently outlined and reflected on the advancement of optical fibre chemical and biosensors from various perspectives [31,32,44,45,46,47,48]. There are abundant possibilities and potential for fabricating highly effective optical fibre ethanol sensors in view of the rapid development of this technology and related functional materials [31]. This article focuses on a review of recent optical fibre sensing developments for ethanol measurement in aqueous solutions, including a perspective on their design and response characteristics. A comparison of their respective performance is provided, which points to their applicability for use in current and future full-scale industrial measurement systems. The continuously developing needs of real time measurement and the emergence of the internet of things (IoT) sets the background to this article, which is intended to inspire and focus further research and wider utilization of these sensors in the commercially significant industrial and medical sectors.

## 2. Optical Fibre Sensing Methods

A basic optical fibre sensor system includes a light source, a sensor element and a detector. An optical fibre sensor interprets a change in the target analyte as a modulation of an optical signal and outputs the modulated optical signal for transmission to humans, computers or other devices in real time, sometimes over long distances [31]. Optical fibre sensors are generally made of glass or plastic optical fibres. A detailed description of the working principles of optical fibre sensors is widely available in the existing literature [49,50,51] and is beyond the scope of this review.

Conventional optical fibres used in communications and sensing have been historically based on a classical solid core and solid cladding structure. However, there have been some exceptions to these, e.g., UV-transmitting fibres [52]. Microstructure Optical Fibres (MOFs) represent a relatively recent development in optical fibre technology. Microstructured fibres (MSFs) are different from conventional optical fibres in their principle of light propagation. Propagation is supported either by a difference in the effective RI between the solid core and the surrounding honeycomb structure or via the photonic bandgap phenomenon in the case of hollow-core MOFs (where the solid core is replaced by a hollow). There are many variations on this structure, e.g., suspended-core MSFs or hollow-core MSFs, but a detailed description of these is beyond the remit of this review and further details are widely available in the existing literature [53].

There are various techniques available to realise the sensing function of these fibres by enhancing the evanescent field. This includes adopting variations in fibre geometry or shape in the sensing region including bending, polishing, etching, tapering and adding femtosecond laser-inscribed gratings. These techniques have been explored to measure ethanol concentration depending on the sensitivity, selectivity and specific application requirements. They can be categorised as absorption-based sensors, interferometric sensors, fibre grating sensors and plasmonic sensors. The following section includes a review of these sensor categories including tabular-based comparisons of design and response characteristics.

### 2.1. Absorption-Based Sensors

**Figure 2:**
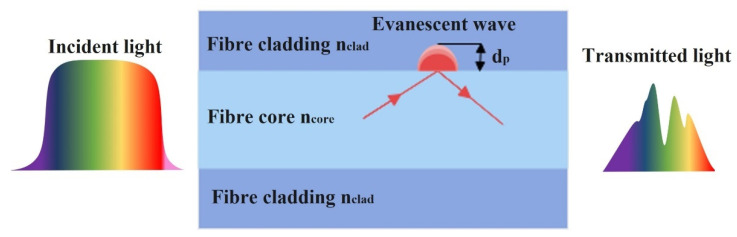
Schematic diagram of evanescent wave principle in absorbance-based optical fibre sensors.

Absorption-based optical fibre ethanol sensors can be described using attenuated total reflection theory. This phenomenon is based on the attenuation of light due to the absorption of the evanescent wave field in the surrounding medium of the fibre or due to light absorption in sensitive material surrounding the fibre. A small amount of light energy is lost from the core during each TIR occurrence. As a result, variations in ethanol concentration modulate the light intensity arriving at a distal detector. An evanescent wave is generated when the energy of the propagating light signal is present in the cladding or other surrounding medium, which creates its own electromagnetic field in that region, as shown in Figure 2. 

The evanescent field strength of a standard solid core optical fibre, with a core and cladding, decays exponentially as the distance from the core increases and can be expressed as:

(1) $$\;I_{x}=I_{0}e^{-x/d_{p}}\;$$

where *I*_0_ is the intensity at the interface, *I_x_* is the optical intensity at a perpendicular distance of *x* from the interface and *d_p_* is the penetration depth, which is the distance at which the intensity drops to 1/e of its initial value at the core and cladding interface. The penetration depth *d_p_* can be expressed as:

(2) dp=λ2πn1sin2θ−(n2n1)2 

where *λ* is the wavelength of the incident light in vacuum, *θ* is the incident angle of the light (in case of MMF), *n*_1_ and *n*_2_ are the RI of the fibre core and cladding, respectively. It can be seen from Equation (2) that the penetration depth is dependent on the incident angle of the light at the interface (*θ*), and the contrast between the core and cladding RIs. The penetration depth increases with decreasing incident angle and contrast between the core and cladding refractive indices. This can be achieved by modifying the fibre geometry or removing the fibre cladding and coating the core with a higher RI sensitive material. Equations (1) and (2) show that the attenuation of the transmitted light signal is dependent on the surrounding RI of the fibre core. Geometrical modification of the fibre with sensitive coatings such as nanostructures greatly enhances the interaction of the evanescent field with its surroundings and, hence, increases the sensitivity of the ethanol sensor. An absorption-based optical fibre sensor is characterised in terms of the change in intensity (or wavelength distribution of the intensity) at the receiver with the change in ethanol concentration. This change in absorption can manifest as a pure intensity change (no spectral modulation) or as a change in the transmitted light spectrum, e.g., through the appearance of dips or absorption edges in the received spectrum. Many geometrical deformations of optical fibres have been reported in the literature to enhance the sensitivity of absorbance-based ethanol sensors such as U-shaped, coil-shaped, meander-shaped and tapering sensors as well as a combination of bending and tapering sensors [35,41,54,55,56].

Based on the concepts outlined above, several absorption-based optical fibre ethanol sensors have been proposed. In 2004, MacDonald et al. used tapered chalcogenide glass fibres to record the spectra of water–ethanol mixtures using infrared fibre evanescent wave spectroscopy (FEWS). Te2As3Se5 (TAS) glass rods were drawn into a fibre with a diameter of around 400 micrometres and the fibre was tapered to a diameter of 200 micrometres along its circumference. The agreement between calculated and experimental spectra suggested that the proposed measurement approach was more accurate than other existing techniques investigated [57]. Fabian et al. demonstrated U-bent, coil-shaped and meander-shaped sensors for ethanol and methanol measurement in fuel cell applications using quartz/quartz fibres of 600 µm diameter [54,58]. The meander shaped sensor exhibited higher sensitivity in comparison to U- and coil-shaped sensors, owing to a higher concentration of bends in the sensing area. The meander-shaped sensor allowed for the measurement of ethanol concentrations of less than 0.2% in water at 650 nm using the visible wavelength region (VIS). Sensors were characterised for ethanol concentrations ranging from 0 to 10% [54]. Luo et al. used a solid-core photonic crystal fibre (PCF) with large holes to measure ethanol concentration [49] and could detect ethanol solutions of various concentrations, ranging from 0.1 to 1%. It exhibited a sensitivity of 0.461 dB per % change in concentration. The optical properties of the PCF were also studied using the finite element method for potential use in future biosensing applications [59]. 

**Figure 3:**
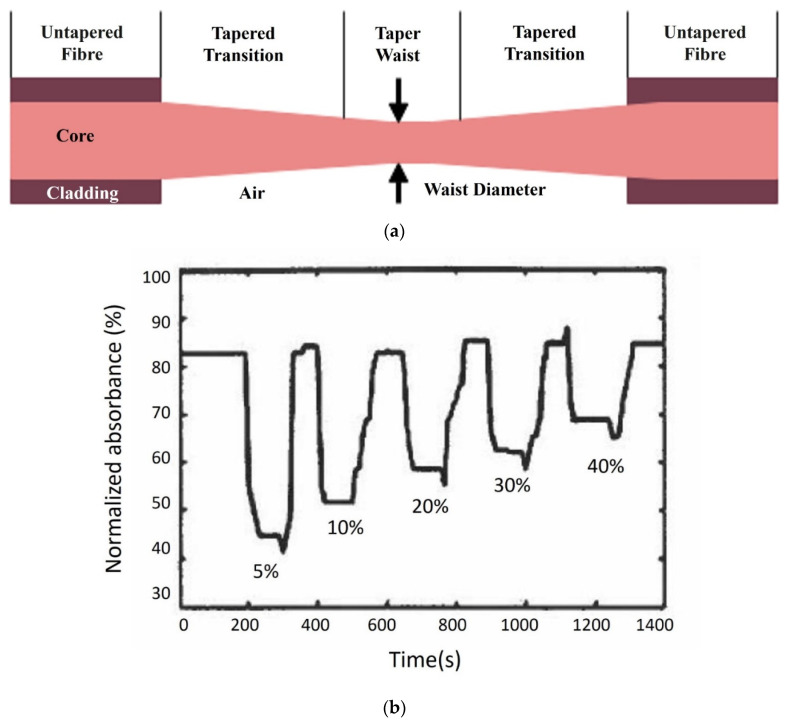

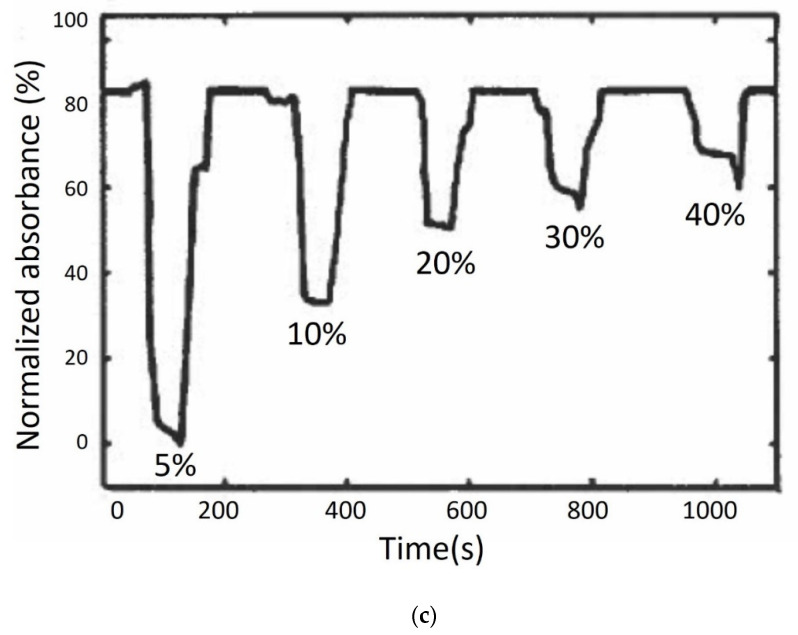
(**a**) Schematic diagram of tapered optical fibre ethanol sensor. Dynamic response of (**b**) graphene- and (**c**) GO-coated tapered optical fibre sensors for different ethanol concentrations in water. Reprinted, with permission, from Springer Nature: Optical Review Ref. [60]; copyright 2015.

Girei et al. used a tapered silica multimode optical fibre sensor design (the generic form of which is shown in Figure 3a) to measure ethanol concentration in water. The sensor had a taper length of 20 mm and a waist diameter of 40 μm [35] and responded linearly and reversibly towards aqueous ethanol in the range of 5–40%, and the typical response and recovery time were 14 s and 27 s, respectively. In a follow-on publication, the authors compared the characteristics of graphene and graphene oxide (GO) as the coating materials in the case of multimode tapered optical fibre sensors [60]. The results showed that the GO-coated tapered sensor had higher absorption magnitude changes compared to the graphene-coated tapered sensor, owing to the higher surface area in the GO film, as shown in Figure 3b,c. The GO-coated sensor exhibited higher sensitivity for ethanol concentration in the range of 5% to 40% and a faster recovery time with a similar response time as the graphene-coated sensor [60]. Gao et al. fabricated a U-bent plastic clad silica fibre which was coated with GO to enhance the sensitivity [61]. The absorption spectra and the dynamic absorption reaction of the U-bent optical fibre sensor with and without GO film were compared for aqueous ethanol with concentrations ranging from 5% to 100%. The sensor with the GO film exhibited superior resolution and sensitivity with shorter response and recovery times, which were in the range of 1–2 s. Molybdenum disulfide (MoS_2_) film is considered to be more easily biomodified, chemically stable and requires low synthesis temperature than graphene. Li et al. proposed a U-bent tapered multimode fibre (TU) coated with a molybdenum disulfide (MoS_2_) film (MoS_2_@TU fibre EWA sensor) [55]. A silica glass MMF of 62.5/125 μm core/cladding diameter was initially tapered over a 3 mm length with a waste diameter of 50 µm and then bent to form a U-bent structure. A comparison between bare U-bent fibre, U-bent fibre with MoS_2_ film and TU fibre with MoS_2_ film was made for the measurement of ethanol concentrations in water ranging from 0 to 100%. The MoS_2_@TU fibre EWA sensor exhibited a higher change in absorption, i.e., 34% and higher sensitivity, i.e., 0.34 (change in absorption ∆A%/change in concentration ∆C%) in comparison to the bare and U-bent fibre with a MoS_2_ film, which was stated as 0.22. This sensor was also found to be sensitive towards adenosine which the authors’ state make them an interesting candidate for bio-sensing applications.

**Figure 4:**
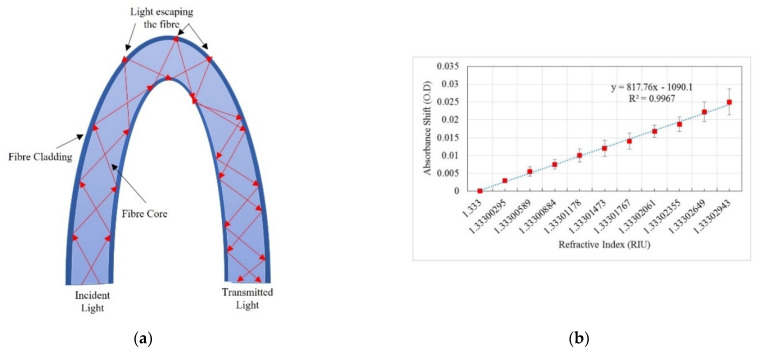
(**a**) Schematic of U-bent POF absorbance-based sensor. (**b**) Absorbance shift response versus change in ethanol concentration. © 2018 IEEE. Reprinted, with permission, from Ref. [41].

**Figure 5:**
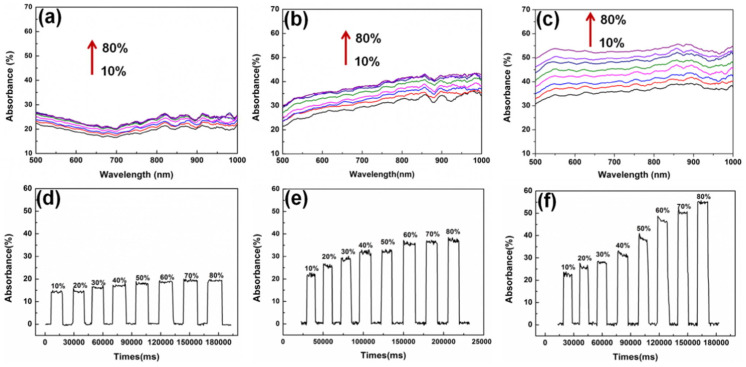
Absorbance spectra for 10% to 80% of ethanol concentration using (**a**) WS_2_@U-bent fibre for 30 s reaction time, (**b**) AuNPs/WS_2_@U-bent fibre for the 30 s reaction time and (**c**) AuNPs/WS_2_@U-bent fibre for the 60 s reaction time. The graphs (**d**–**f**) represent temporal responses at 800 nm wavelength based on the (**a**), (**b**) and (**c**) spectra, respectively. Reprinted from Ref. [63], copyright 2018, with permission from Elsevier.

A Plastic Optical Fibre (POF) with 1 mm overall diameter was investigated for use as an ethanol concentration sensor by Memon et al. [41,62]. A U-bent POF ethanol sensor for real-time sensing in biofuel production applications was designed as shown in Figure 4a. The sensor was characterised for measurement in the initial stages of bioethanol production, i.e., in the concentration range of 0.005% to 0.05% (*w*/*w*) in water. In the absence of reported literature on the minute RI changes, they theoretically estimated the RI values for the same range using Lorentz–Lorenz equations and related them to the absorbance response of the sensor. The sensor exhibited a resolution of 10^−7^ Refractive Index Unit (RIU) with 99.76% linearity in the response. The sensitivity and limit of detection (LOD) were found to be 817.76 Optical Density/Refractive Index Unit (O.D./RIU) and 9.2 × 10^−7^ RIU, respectively, as shown in Figure 4b. Khalaf et al. showed carbon nanotubes (CNT) to be sensitive to aqueous ethanol solutions in the range of 20% to 100% when they were coated on an unclad multimode POF (UCPOF) using a drop-casting technique [56]. The GO nanomaterial was also synthesised and used as a sensing layer to obtain a comprehensive view of the CNT-based sensor’s performance. Experiments showed that the CNT-based UCPOF sensor exhibited a four-fold higher sensitivity to aqueous ethanol than the GO-coated sensor. The UCPOF sensors based on CNT were also characterised using other Volatile Organic Compounds (VOCs) and they achieved a higher selectivity for ethanol than other analytes tested. The presence of gold nanoparticles (AuNPs) grown in situ on tungsten disulfide (WS_2_) resulted in an enhanced optical coupling between the evanescent wave and the AuNPs, making a U-bent optical fibre EWA sensor significantly more sensitive to environmental changes [63]. Ethanol and NaCl were used to illustrate the sensing capabilities of the proposed AuNPs on WS_2_@U-bent optical fibre EWA sensors. The absorption spectra were captured for the WS_2_@U-bent fibre, AuNPs/WS_2_@U-bent fibre for the reaction time of 30 s and AuNPs/WS_2_@U-bent fibre for the reaction time of 60 s for the ethanol concentration in the range of 10–80%, as shown in Figure 5. The AuNPs/WS_2_@U-bent fibre sensor for the reaction time of 60 showed the highest sensitivity of 0.65 for detecting ethanol solution (∆A/∆C) [63]. 

The examples illustrated above demonstrate the feasibility of optical fibre-based methods based on absorption for aqueous ethanol concentration measurement. These examples are further summarised in Table 2 and classified according to details of structure design, fibre type, sensitive coating, light sources and detectors, sensitivity (S), resolution (R), measurement range and type of application. Table 2 shows that absorption-based optical fibre ethanol sensors are highly versatile in terms of their ease of design and implementation using robust materials. However, deformation of the fibre to improve the sensitivity, e.g., through tapering can make the sensor fragile and susceptible to breakage. Low selectivity is also a concern with sensors in the absence of a selective material coating. 

### 2.2. Externally Modified Interferometric Sensors

**Figure 6:**
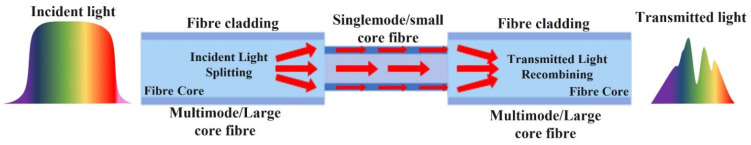
Schematic diagram of a multimode–singlemode–multimode optical fibre sensor [70].

Optical interferometry is an established method that has achieved high-accuracy measurements in a variety of applications such as temperature and pressure measurement, refractive index measurement, growth rate in crystallization and surface metrology [64,65]. Two-beam amplitude-division interferometry is commonly used for sensing applications and can be described as an interference pattern produced by the recombination of two light beams of the same frequency, constant phase difference and identical direction [66]. The resulting intensity can be expressed as follows:

(3) $$I=I_{1}+I_{2}+2\sqrt{I_{1}I_{2}}\;\cos\emptyset \;\;$$

where *I*_1_ and *I*_2_ are the intensities of two light beams and Ø is the phase difference between *I*_1_ and *I*_2_. The phase difference Ø is given by:

(4) $$\emptyset =\frac{N\pi nL}{\lambda }\;$$

where *N* is an integer and its value is dependent on the type of interferometry, *n* is the RI of the transmitting medium and *L* is the optical path length over which the phase change occurs. The main interferometric techniques used for optical fibre ethanol concentration measurement include Fabry–Perot, Mach–Zehnder and Michelson. Generally, changes in a sensing layer or film coated on the outside of the fibre in one of the interferometer’s arms are monitored with the change in ethanol concentration. A number of different sensing film materials have been investigated for this purpose. Due to the physical absorption/adsorption of ethanol molecules, the sensing layer generally changes one or more of its physical properties (e.g., thickness or volume) or optical properties (e.g., RI) causing a change in optical path length experienced by different wavelengths and, hence, a phase shift in the output spectrum. However, interferometric techniques have also been explored without any sensing film with promising outcomes [36,67,68,69]. A schematic diagram of a multimode–singlemode–multimode (MSM) sensor is shown in Figure 6.

Mudhana et al. proposed a single-arm common-path interferometer optical fibre structure with a cavity (singlemode fibre) length of 1 mm [67]. A unique large radius bifunctional PCF lens eliminated the requirement for two arms as is the case in a traditional interferometer. This specifically developed PCF lens served as both the reference arm’s reflector and the sample arm’s collimator. The bifunctional lens tip and a micro-mirror were encased in a glass tube to create the sensor element for detecting the RI of liquids. A hole was drilled in the glass tube to allow the liquid sample to freely flow in and out of the cavity. The RIs of acetone, ethanol and distilled water were measured using the probe and an analytical resolution of 2.6 × 10^−5^ RIU was achieved. The authors further suggested replacing the sensor element with a reflection-coated fibre to reduce the sensor’s size. Yuan et al. developed an inline optical fibre Michelson Interferometer (MI) by creating, on a sample-by-sample basis, a step structure at the tip of a standard singlemode optical fibre (Corning SMF-28) with a core/cladding diameter of 8.2/125 µm [71]. A femtosecond laser was used to micromachine a step structure which split the reflected light path into two paths. Both temperature and RI sensing were successfully demonstrated, and ethanol was specifically used for the measurements in the range of 0–50% in liquid solutions. A nonlinear relationship between wavelength shift and concentration was observed and it was qualitatively related to the Langmuir adsorption model. This design showed a limitation of low sensitivity for RI measurements. The measurements were also recorded rather than monitoring a continuous change in solution concentration. 

**Figure 7:**
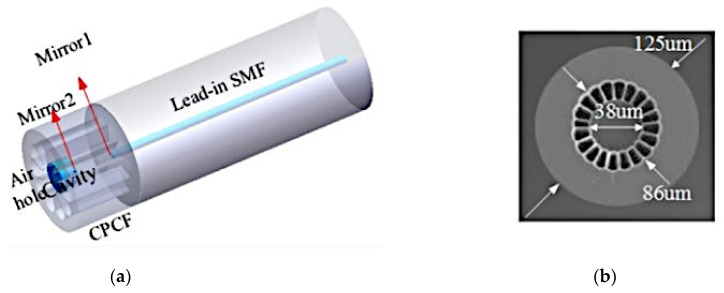
(**a**) Schematic structure of the concave-core PCF FPI Sensor. (**b**) Microscopic cross-sectional view of the CPCF. Reprinted/adapted with permission from [36]; © The Optical Society.

**Figure 8:**
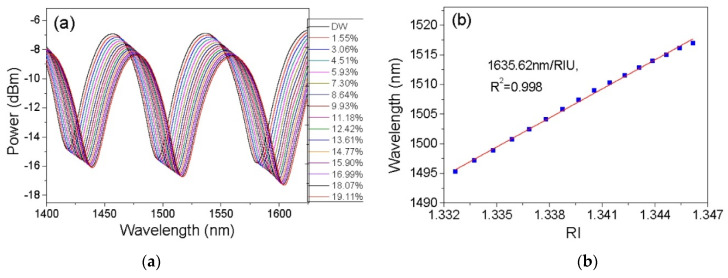
(**a**) Reflection spectra of CPCF PFI sensor when exposed to ethanol–water concentrations from 0 to 19.11%. (**b**) Relationship between the dip wavelength centred around 1500 nm versus ethanol–water solutions’ RI changes. Reprinted with permission from [36]; © The Optical Society.

Wu et al. proposed an alternative fibre inline interferometer based on an open-cavity Fabry–Perot Interferometry technique [68]. The sensor involved fusion splicing a C-shaped thin fibre between two standard singlemode fibres. In this case, the C-shaped fibre served as an open cavity, which allowed the gap between the two single-mode fibres to be filled by water–ethanol solutions in the RI range of 1.33 to 1.36. The performance of the sensor was also tested by changing the cavity length where a shorter cavity length was observed to increase the measurement range and detection limit. This sensor design demonstrated a maximum sensitivity of 1368 nm/RIU at 1600 nm wavelength with a linear response of R^2^ = 0.996 and very low temperature cross-sensitivity of 3.04 × 10^−7^ RIU/°C. The main limitation of this sensor design was that the C-shaped fibre is not a commercial product, and its production is complicated and expensive. Photonic crystal fibres have also been explored for open-cavity FPIs. Tian et al. explored the use of a concave-core photonic crystal fibre (CPCF) as an open-cavity FPI microfluidic RI sensor with a fast reaction time [36]. A small section of multimode PCF was cleaved with an axial tension to create the CPCF and the shallow concave core formed a Fabry–Perot cavity of very short length which broadened the measurement range. The multiple air holes in the CPCF acted as microfluidic channels, allowing liquid samples to be effectively delivered into and out of the sensor. Figure 7 shows the schematic of the CPCF FPI sensor structure and cross-sectional view of the CPCF. This sensor design was characterised using 0 to 19.11% ethanol in distilled (DI) water and exhibited a sensitivity of 1635.62 nm/RIU at 1500 nm wavelength as shown in Figure 8. The measurement response times for this sensor design were less than 23 ms and 359 ms for pure ethanol and distilled water, respectively.

Muri et al. used a hydrogel half-sphere on the fibre end face to form a low-finesse Fabry–Perot cavity [72]. They demonstrated the quality of interferometric and Localised Surface Plasmon Resonance (LSPR) characteristics of gold nanoparticles (GNP) fixed in an acrylamide hydrogel of the fibre end-face as a first step for a combined interferometric and LSPR-based optical fibre sensor. The free spectral range (FSR) and LSPR were monitored as a function of hydrogel swelling degree with increasing ethanol concentration from 30% to 50%. A similar sensor also demonstrated promising results for medical applications in terms of label-free and selective sensing of biomolecules [72].

Multimode interference (MMI) devices have also attracted significant attention due to their ease of fabrication. Rodriguez-Rodriguez et al. proposed such an MMI produced by splicing a short length of MMF between two standard singlemode fibres for monitoring the quality of gasoline/ethanol blends for flexible-fuel vehicles (FFV) [73]. A coreless MMF of 125 μm diameter was used as the MMF part of the sensor. The MMI sensor was used for making RI measurements using various chemical solutions and exhibited a sensitivity of 133.65 nm/RIU for a 1.318 to 1.373 RI range, and a sensitivity of 390.88 nm/RIU for a 1.373 to 1.420 RI range. Solutions ranging from E50 ethanol–gasoline blend to pure G87 gasoline were used for characterization. Reducing the amount of ethanol produced a red shift in the observed spectrum due to the higher RI of pure G87 Gasoline. Marfu’Ah et al. proposed an alternative configuration of MMI based on splicing a short length of singlemode fibre between two multimode fibres as a multimode–singlemode–multimode (MSM) structure where part of the energy of the core mode is transferred to cladding modes [74]. Experiments were performed using the MSM sensor with and without a novolac resin coating. The sensor with the novolac coating indicated higher sensitivity in comparison to the sensor with no novolac resin coating in alcohol–water and alcohol–sugar solutions in the range of 0 to 10%. The sensitivity of the MSM sensor with the novolac resin coating was measured to be 0.028972 dBm per % *v*/*v* and 0.005005 dBm per % *v*/*v* for alcohol–water solutions and alcohol–sugar solutions, respectively. 

**Figure 9:**
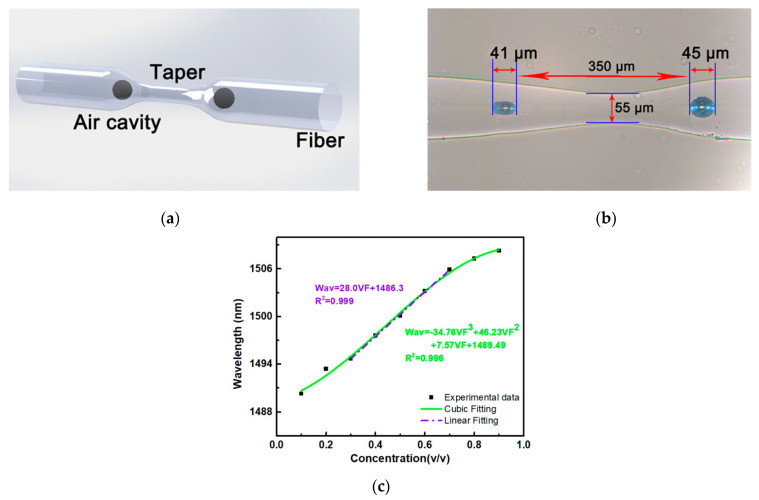
Fibre taper-based MZI: (**a**) schematic diagram, (**b**) microscopic image and (**c**) shift in the wavelength of the dip near 1485 nm versus volume fraction of the aqueous ethanol solution. Reproduced from Ref. [43].

Liao al. investigated the use of an optical fibre taper-based Mach–Zehnder interferometer (MZI) configuration for ethanol concentration measurement [43]. The MZI was based on a taper and two inner air bubbles created using femtosecond laser processing as well as a combined splicing-tapering technique as shown in Figure 9a. Figure 9b is a microscope image of the structure. A non-linear relationship between the ethanol concentration and dip wavelength was observed when aqueous ethanol solutions of concentrations ranging from 10% to 90% were tested. A linear response was observed in the 30% to 70% ethanol–water concentration range, as shown in Figure 9c, with a sensitivity of 28 nm/vol or 592.8 nm/RIU. Recently, Zhou et al. proposed a Michelson Interferometer optical fibre probe for RI measurement, where the main highlight was the capability of temperature compensation of liquids during the RI measurement [69]. Zhou et al. developed a cavity using femtosecond laser machining located very close to the end of a cleaved fibre tip. The fibre tip was cleaved at a 45° angle with respect to the fibre axis. When this sensor was exposed to air it created a Fabry–Perot cavity and behaved as a Michelson interferometer when exposed to liquid. This sensor was not directly interrogated for ethanol as the liquid of interest. However, it was characterised in terms of the RI range for which experimental data matches with typical ethanol RI values. The temperature dependence for ethanol, methanol and water were measured at an interrogating wavelength of 1550 nm, where the measured results agreed well with experimental data from other researchers. However, there was a considerable change in the thermo-optic coefficient of methanol with a theoretical estimate. This sensor exhibited a sensitivity varying from 885.437 to 1067.525 nm/RIU when the RI varied from 1.3166 to 1.4346. 

The performance of the Interferometric Optical Fibre Ethanol sensors referenced above is further summarised in Table 3 in terms of sensor design, fibre type, sensitive coating, light sources and detectors, sensitivity (S), resolution (R), measurement range and type of application. From the above examples, it must be emphasised that some interferometric techniques require costly, precise and sometimes delicate fabrication process steps such as femtosecond laser machining. On the other hand, multimode interferometers are relatively easy to implement and have simple structures that include flexible design, with a tradeoff of difficult prediction of the output due to the non-periodicity of the spectrum, which can give rise to a highly complex signal.

### 2.3. In-Fibre Grating Sensors

**Figure 10:**
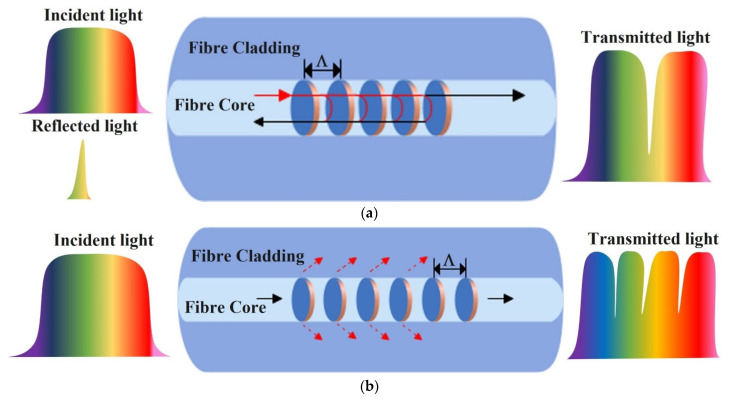
Schematic diagrams of (**a**) FBG sensor and (**b**) LPG sensor.

A grating in an optical fibre is formed as a periodic variation in the core RI. Fibre gratings are generated as spatial phase gratings in the core and are formed using the photosensitivity of the fibre materials. General fibre gratings can be classified into two types based on their physical structure (or grating period): Fibre Bragg gratings (FBG) with grating periods in the order of 1.5 µm and Long Period gratings (LPG) with grating periods in the order of 100 µm. Light at a certain wavelength can couple to a backward propagating fundamental mode or a forward propagating cladding mode depending on the grating type. As shown in Figure 10a in the case of an FBG, there is coupling between a forward and backward propagating mode over a narrow band of selected wavelengths only, which creates a peak in the reflection spectrum, giving rise to a specific Bragg-reflective wavelength *λ_B_*, given as [75]:

(5) $$\lambda_{B}=2n_{eff}\Lambda \;$$

where *Λ* is the grating period and *n_eff_* is the effective RI. Normal FBGs formed in standard SMFs are inherently insensitive to the RI of the surrounding environment. FBG-based optical fibre ethanol sensors are either developed by reducing the cladding diameter by etching to enhance interaction of the core modes with the surrounding refractive index (SRI) or by coating a sensitive material on the cladding or both. As FBGs are inherently sensitive to strain within the fibre, many FBG sensors are fabricated to respond to a change in volume of the coating material when exposed to the measurand, e.g., ethanol solution, which in turn causes a shift in the reflected spectrum due to a change in the grating period [76].

In the case of LPGs, optical coupling is established between the forward propagating core mode to the cladding modes, which takes place at certain wavelengths only. The pattern of the coupling variation with wavelength is repetitive over the spectral transmission range of the fibre which gives rise to a classical photonic bandgap behaviour of these devices, as shown in Figure 10b. The grating period is defined as distance between the points where the core and cladding modes are in phase. The phase-matching condition determines the resonances of the long-period grating, as given below [75]:

(6) $$\lambda =\Lambda \left(n_{eff}^{co}-n_{eff}^{cl}\right)\;$$

where *Λ* is the grating period and $n_{eff}^{co}$ and $n_{eff}^{cl}$ are the effective refractive indices in the core and cladding, respectively. The interaction between the core and cladding modes in the case of an LPG is much stronger than in the case of an FBG and, therefore, LPG-based optical fibre sensors exist with and without coatings.

In 2003, Keith et al. investigated the performance of bare (uncoated) LPG fibre sensors fabricated without any sensitive material for measurement of standard RI solutions and mixtures of methanol and ethanol [77]. Initially, they tested the sensor’s performance using standard RI mixtures, but also measured the temperature dependence of the sensor and repeatability of their results. Having established the fundamental sensor response to RI and temperature, Keith et al. examined varying the solution composition from 100% methanol through 50/50 methanol/ethanol to 100% ethanol at a set temperature of 20 °C. Variations in the measured wavelengths in solutions produced a relative standard deviation of only 0.046 and 0.029 percent for methanol and ethanol, respectively, indicating excellent repeatability. With this method, unknown combinations of mixtures may be precisely determined. The observed accuracy was in the 0.5–1% range but producing repeatable results was a major issue. Raikar et al. produced gratings on one end of a singlemode Ge-B co-doped photosensitive fibre using the phase mask technique and then etched the region where the gratings were formed for ethanol concentration measurement [78]. The sensor was characterised for ethanol concentration ranging from 0 to 50%, where red wavelength shift was observed with increasing ethanol concentration and exhibited a sensitivity of 0.002 nm/%.

**Figure 11:**
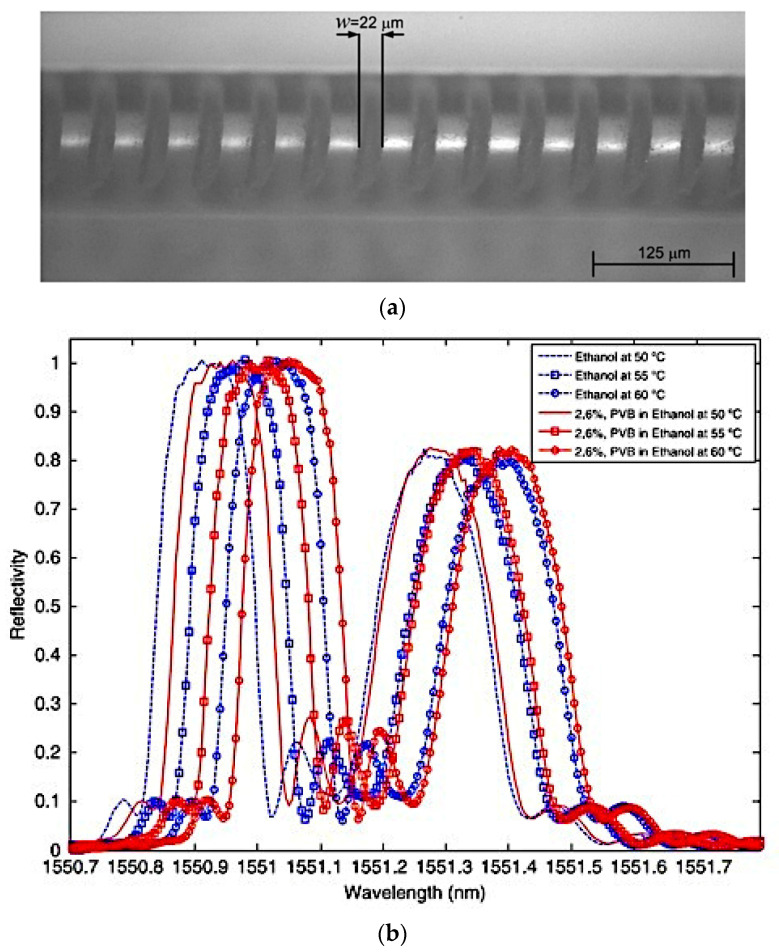
(**a**) Micro-grooved structure fabricated on the fibre. (**b**) Reflection spectra of micro-groove-structured FBG sensor for ethanol concentration and different concentrations of PVA in ethanol at different temperatures. Ref. [42]; © IOP Publishing. Reproduced with permission. All rights reserved.

Alemohammad et al. proposed a femtosecond (FS) laser micromachining fabrication process to inscribe micro-grooves in the cladding of the optical fibre, thereby incorporating FBGs for effective coupling of the propagating core modes to the surrounding medium [42]. A microscope image showing the on-fibre fabricated structures is shown in Figure 11a. The prepared FBG sensors were immersed in solutions of 2.6% and 4.8% polyvinyl butyral (PVB) in ethanol for concentration measurement along with their thermal responses, for which reflection spectra are shown in Figure 11b. The findings demonstrated enhanced sensitivity to the concentration of the surrounding medium and these micromachined surface-modified FBG sensors may be used for simultaneous concentration and temperature sensing in chemical and biological liquids. 

Possetti et al. investigated a refractometric sensor based on LPG fibres as a tool for the industrial sector of fuel mixtures or ethanol–gasoline blends [79]. Standard single-mode fibre SMF-28 was used for writing an LPG with a length of 21.6 mm and period of 540 µm. The sensor was tested for measuring ethanol in gasoline in concentrations ranging from 0 to 100% by introducing the samples into a glass cell. The LPG sensor indicated an average sensitivity of 43 pm/% for ethanol concentrations ranging from 20% to 40%, which is the range of significance for the Brazilian industrial sector. The combined and expanded uncertainties for the LPG refractometric sensor were found to be smaller than those for an Abbe refractometer, which were 0.7% and 1.7%, respectively. The same group later proposed an encapsulated LPG version for better device stability, protection from external damage and reproducibility of the LPG fabrication during multiple production cycles [80]. The encapsulation device comprised a quartz tube and a steel shield and was deemed to have minimal effect on LPG sensitivity. Both systems had a sensitivity of 10 nm/RIU for aqueous ethanol solutions with RIs ranging from 1.33 to 1.36, while samples with RIs ranging from 1.42 to 1.44 (gasoline, turpentine and kerosene) indicated a sensitivity of 102 nm/RIU. The authors concluded that it significantly improved the standard deviation in results, being as good as 58% for ethanol and 78% for gasoline, as compared to the non-encapsulated version [79].

The performance of an etched FBG constructed in various configurations to function as a refractometric sensor was analysed, for water–ethanol mixtures ranging from 0 to 100%, by Coradin et al. [81]. Two wet-etched FBGs with Bragg wavelengths close to 1300 nm and 1500 nm were used and showed sensitivities of 6.5 ± 0.2 nm/RIU and 2.9 ± 0.2 nm/RIU at 25.0 ± 0.5 °C, respectively. They also investigated the effect of the ambiguous relationship between RI and ethanol content in a mixture above a concentration of 80% *v*/*v*. The sensor setup, based on the etched FBG1300, had a greater RI sensitivity compared to the FBG1500 version and showed combined uncertainties of 2.8% *v*/*v* and 7% *v*/*v* below and above the 80% *v*/*v* of ethanol concentration, respectively. Arasu et al. investigated an absorbance-based FBG ethanol sensor by coating thin films of gold of various thickness on clad FBGs [82]. They compared the absorbance responses of standard uncoated SMF, coated SMF, uncoated FBG and coated FBG. Sensors were coated with 35 nm, 40 nm and 50 nm thin gold films and it was observed that increasing the thickness of the gold layer increased the absorbance of sensor. Sensors were characterised for ethanol–water mixtures for ethanol concentrations ranging from 0 to 99.7%. FBG sensors with 50 nm gold coating demonstrated good repeatability and higher sensitivity. The response and recovery time were observed to be approximately 20 and 35 s, respectively. 

**Figure 12:**
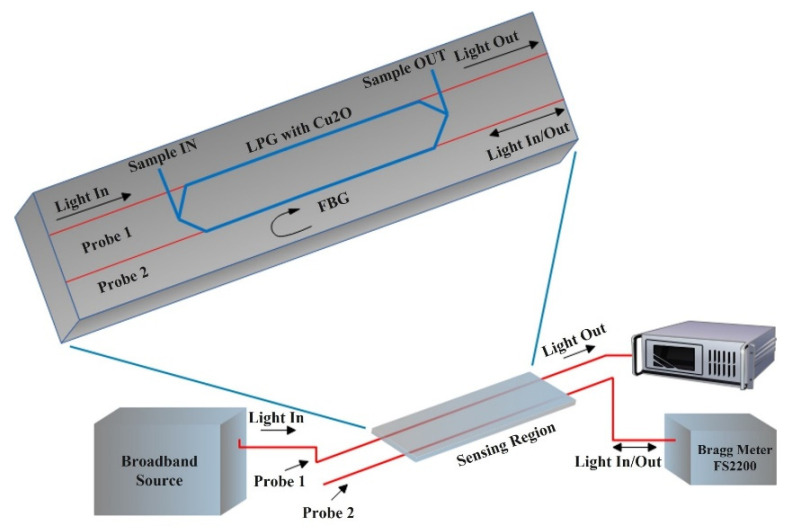
Cuprous oxide-coated LPG- and FBG-based optical fibre sensing setup with a PMMA-based flow cell for sample inflow and outflow. Adapted from Ref. [37].

An optical sensing method based on a combination of an FBG and an LPG coated with cuprous oxide (Cu_2_O) was reported by Monteiro-Silva et al., where the LPG was used for ethanol quantification in ethanol–gasoline mixtures and the FBG was used for monitoring temperature [37]. They incorporated a PMMA-based flow cell for handling the sample in and out flow as well as providing access for the light input and output fibres of the two sensing probes as illustrated in Figure 12. The authors included results for spectra of the LPG probe when operated in air, pure ethanol, pure gasoline (no ethanol) and ethanol–gasoline mixtures of 1.5 to 30%. A Cuprous oxide-coated LPG indicated a higher sensitivity of 0.76 ± 0.01 nm/% *v*/*v* (wavelength shift response) and 0.125 ± 0.003 dB/% *v*/*v* (power attenuation response) for ethanol concentration ranging from 1.5% *v*/*v* to 30% *v*/*v* in ethanol–gasoline mixtures in comparison to uncoated LPGs. The resulting resolution was determined as 5.1 × 10^−4^ RIU and the authors state this optical fibre sensing system provided good performance whilst avoiding the use of expensive gas chromatography techniques to determine the ethanol–gasoline sample compositions [37]. Bui et al. presented a novel optical sensor design based on dual FBGs integrated in an erbium-doped fibre ring laser structure for measuring wavelength shift without the need for a spectrometer as a detector [83]. They used one FBG as a reference and an etched FBG as a sensing probe. The principle was based on the reference FBG sweeping over the entire spectrum of the etched FBG. This sensor design demonstrated identification of 0–14% *v*/*v* mixing ratios of ethanol and/or methanol in gasoline RON 92 (research octane number) with potentially high repeatability, accuracy, quick response and a limit of detection of 1.5 × 10^−4^ RIU. They also experimented for nitrate—water samples. This design therefore demonstrated high potential as an inexpensive biochemical sensing method. 

Aristilde et al. investigated a tilted FBG (TFBG) for measurement of gasoline-adulteration avoiding the need for additional fibre etching when using FBGs [84]. The sensor measured ethanol concentration in gasoline–ethanol solutions using the convoluted response of two Bragg gratings as input for a TFBG. They interrogated responses of ethanol concentration from 0 to 60% in gasoline using two OSAs and photodetectors where the cladding modes were accessed to measure the change in RI surrounding the TFBG and the core modes were interrogated for temperature variations. It was estimated that the sensor can detect ethanol concentration changes of 1.5% in gasoline–ethanol mixtures and distinguish temperature variations of 0.5 °C. Kumar et al. recently utilised a GO-coated Etched FBG (EFBG) sensor for ethanol measurement in petrol [85]. When compared to an un-coated EFBG, the GO-coated etched FBG (EFBG) demonstrated a ten-fold increase in ethanol sensitivity. This sensor was based on measurement of changes in the intensity of the reflection spectra as the percentage of ethanol in petrol was varied from 0 to 100%. The ethanol proportion in the fuel was detected down to 0.5%, which was stated as being better than the standard EFBG-based purely on wavelength shift measurements. This sensor design exhibited a sensitivity of 0.18 dB/percent concentration and negligible temperature sensitivity within 5 °C of the external environment’s value. 

The sensors discussed in this section have been used for general and gasoline quality monitoring applications. It has been observed that FBGs are not inherently sensitive to chemical changes in the surrounding environment and require etching or a reduction in the cladding to become sensitive to surrounding ethanol changes. This makes them fragile and less robust, and temperature cross-sensitivity is a matter of concern. However, different configurations such as tilted FBGs can make them sensitive to the surrounding RI whilst maintaining a robust structure. On the contrary, LPGs do not require etching or deformation of the fibre and provide good sensing resolution. LPGs are inherently sensitive to temperature changes, but some compensation methods have been successfully deployed to overcome this, e.g., using a combined LPG and FBG sensing scheme. Grating sensors often require costly interrogation systems such as spectrum analysers or spectrometers. This obstacle can be mitigated, e.g., using a dual FBG interrogation scheme [83], which may be helpful for developing a future low-cost ethanol sensing system. 

The different configurations of grating sensors including LPGs, FBGs, EFBGs, TFBG, Dual FBGs, Metal-coated FBGs and Micro-grooved FBGs are illustrated in Table 4 which summarises the various configurations in terms of the system attributes, sensor physical/geometrical considerations (e.g., coating type), and their intended application area, sensitivity (S), resolution (R) and measurement range.

### 2.4. Plasmonic Sensors

**Figure 13:**
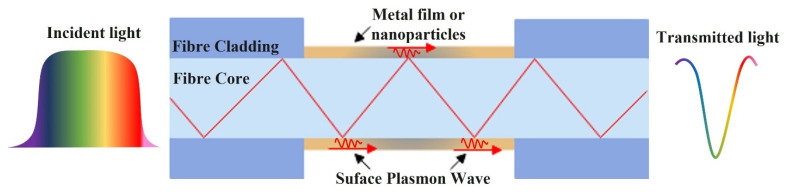
Schematic diagram of plasmonic optical fibre sensor.

Plasmonic optical fibre sensors are based on the Surface Plasmon Resonance (SPR) phenomenon and occurs at a resonance wavelength of the incident light on a metal–dielectric interface due to the collective oscillation of conduction band electrons. This happens due to the excitation of electrons in the metal surface layer by the photons of incident light with a specific angle of incidence. These electrons then travel parallel to the metal surface creating a wave pattern called as Surface Plasmon Wave (SPW) or Surface Plasmon Polaritons (SPPs), as shown in Figure 13. 

Many highly sensitive SPR sensors have been proposed for biochemical analyte testing. Fibre-based plasmonic sensors have drawn growing interest in comparison to classic prism-based plasmonic sensors due to their merits for online monitoring, small size, efficient integration and remote measurement. When light propagates through the fibre core, TIR occurs at the fibre-metal interface which generates an evanescent field as explained earlier in Section 2.1. The wave vector of the evanescent wave is given by [86]:

(7) $$k_{EV}=\frac{\omega }{c}\sqrt{\varepsilon_{o}}sin\theta \;$$

where *ε_o_* is the dielectric constant of the cladding or core (if the cladding is removed) of the optical fibre, *θ* is angle of incidence of the light and *ω* represents the angular frequency of the incident light. The surface plasmon wave vector propagating along the metal–dielectric interface is given by [86]:

(8) $$k_{SP}=\frac{\omega }{c}{\left(\frac{\varepsilon_{m}\varepsilon_{s}}{\varepsilon_{m}+\varepsilon_{s}}\right)}^{1/2}\;$$

where *ε_m_* and *ε_s_* are the dielectric constants of the metal and dielectric medium, respectively. The surface plasmon resonance occurs when *k_EV_* matches with the real part of *k_SP_*.

The fibre surface plasmon may be divided into two categories based on the plasmon mode of transmission: SPR and localised surface plasmon resonance (LSPR). SPR is created by coating a thin metal film on the surface of an optical fibre and the resonance condition that occurs at a specific wavelength generates SPR, leading to a dip/attenuation in the spectrum at the resonance wavelength. LSPR is created by coating metal nanoparticles on the fibre and when the incoming light frequency resonates with the entire vibrational frequency of the conducting electrons in the metal nanoparticles. The light energy is significantly absorbed at this frequency (wavelength) [87]. SPR and LSPR resonant wavelengths change when the surrounding RI of the metal layer changes.

In 1999, Mitsushio et al. proposed an unclad gold-coated optical fibre sensor system for ethanol and ethylene glycol sensing [88]. The results demonstrated the sensor to be capable of operation in the concentration range of 0 to 80% of ethanol in water with a limit of detection of 0.5%. The same sensor was also tested for alcohol content in commercial liquor (Shochu), which indicated good agreement with the labelled ethanol content of 25% *v*/*v* on the bottle. As it is one of the first systems developed for such sensor designs, they used a 632.8 nm He-Ne laser source (Melles Griot V05LHR15) and a discrete lens to illuminate the input of the fibre. These limitations have been improved over the years for SPR sensing systems. A cone micro-tip shaped SPR device was investigated for ethanol and methanol concentration measurement by Kurihara et al. [89]. The sensing technique relied on a polarization rotation in the presence of SPR using 780 nm light obtained from a chopped laser source. They created the cone shape on the step-index optical fibre of a GeO_2_ highly doped silica core using a chemical etching process, which took two hours at room temperature. A gold metal film was then deposited on the cone shape and a groove was subsequently created on the gold metal film by manually scratching using a small screwdriver. The gold film was deposited across the entire end-face of the fibre and was nominally 50-nm thick, but the thickness was estimated to decrease to 13 nm on the tip due to varied deposition and the conical geometry of the tip. The sensor demonstrated a limited resolution of 10^−2^ RIU.

In 2005, Suzuki et al. proposed a dual-colour optical fibre SPR sensor by depositing a silver layer at the fibre tip to create a mirror as well as depositing a gold layer on the surface of the core of a step index silica MMF of 400 µm core diameter [90]. The sensor’s working principle was based on a differential reflectance method. Two optical couplers provided source light from two light emitting diodes (LEDs) of 612 nm and 680 nm that were pulsed alternately in a continuous cycle to the sensor section. The RI changes of ethanol–water solutions ranging from 0 to 50% (1.333 to 1.3616) were observed as being proportional to changes in reflectance at the two wavelengths. The sensor was estimated to have an LOD of 5.2 × 10^−4^ RIU. The possibility of using excitation of SPP was studied by Abrahamyan et al. [91]. They used the tip of a gold-coated conical-shaped optical fibre to measure ethanol concentrations in specific solutions when the tip was dipped in the medium with a particular dielectric permittivity. They performed experiments on a dimethyl sulfoxide and ethanol solution with volume ratio of 0.9. The dielectric permittivity of the solution varied gradually because of evaporation and a corresponding change in the output radiation from the fibre tip was observed as per resonant excitation of SPPs. This sensor design formed the concept for novel optical fibre SPR sensors for chemical sensing with comparable sensitivity to the conventional SPR sensors, i.e., 2 × 10^−4^ RIU.

Mitsushio et al. reported a basic SPR sensor system by depositing a 45 nm Au-film on one half of the core of a MM glass optical fibre and fixing the sensor element and Teflon tubes for fluidic guiding (in and out) of the samples in a glass tube [92]. They investigated the use of different wavelength LEDs (563 nm, 660 nm and 940 nm) to determine the performance of the SPR sensor system. The output intensity versus RI response curves showed dips at different refractive indices for different incident wavelengths, which is indicative of a dependence on the system performance on the source wavelength. The same authors experimented with ethanol solutions in the range of 0 to 50% by volume (% ABV) concentration using a 660 nm red LED light source at 15 °C and produced calibration curves showing resolution comparable to an Abbe refractometer, i.e., 10^−4^ RIU. The calibration curves were used for analysis of measurements on ethanol concentration in eight commercial spirits, where reasonable agreement was observed between the measured and the labelled ethanol content in the spirits. Zhang et al. presented a new approach for surface modification of a tapered optical fibre using star-shaped gold nano particles of 80 to 120 nm in size [40]. They compared two different coupling agents to self-assemble the nano particles, namely: 3-aminopropyltrimethoxy silane (APTMS) and 3-mercaptopropyltrimethoxy silane (MPTMS). A comparison of the transmission spectra of the MPTMS modified fibres revealed a greater sensitivity than the APTMS modified fibres. To demonstrate the sensitivity of the modified fibres, transmission spectra were measured for ethanol and gentian violet solutions. They treated the APTMS and MPTMS modified fibres with t-dodecylmercaptan and observed that APTMS modified fibres became less sensitive to different surrounding media. They further characterised MPTMS modified fibres for ethanol concentrations ranging from 10% to 40%; they showed wavelength and intensity shifts in the spectrum and the sensor indicated a sensitivity of 1190.5 nm/RIU [40]. Hlubina et al. demonstrated two SPR RI sensing configurations, i.e., inline transmission and reflection operating over a wavelength range of 500 to 800 nm provided by a tungsten-halogen broadband light source [93]. They sputtered a 50 nm gold film on both sides of different lengths of unclad multimode fibres for both configurations. In both experimental setups, polarisers were used for transmission configuration which demonstrated the polarization dependence of this scheme. The inclusion of polarisers produced a deep spectral dip. Both sensor configurations were tested for ethanol concentrations ranging from 0 to 80 wt.% and, thus, demonstrated a wavelength shift with increasing ethanol concentration. The reflection sensing scheme resulted in higher sensitivity but also non-linearity at higher concentrations of ethanol. In comparison, the inline transmission configuration, including the polariser, presented lower sensitivity with a more linear response over the range of ethanol concentration changes.

Verma et al. investigated an SPR sensing method combined with functionalization using ethanol-selective enzymes for low ethanol concentration in food and beverages [94]. They first coated the fibre core with 40 nm of silver (Ag) and then immobilised the enzymes using a gel entrapment method. Two different sensor probes were prepared by immobilising the alcohol dehydrogenase (ADH) enzyme only and ADH enzyme with nicotinic acid. The observed resonance wavelength shift for 0 to 10 mM of ethanol concentration in buffer solution was 7.00 nm and 18.33 nm for the Ag/ADH and Ag/ADH/Nicotinic acid probes, respectively. This corresponded to the higher observed sensitivity of the Ag/ADH/Nicotinic acid probe. These sensors were also investigated for their stability, reusability and reproducibility and produced a good response in each case. It was observed that sensors have good sensitivity for low ethanol concentrations as it decreased for higher ethanol concentrations, where the ethanol concentrations ranged from 0 to 10 mM. In follow-up work, a silver/silicon/hydrogel entrapped with ADH-/NAD-coated unclad optical fibre for ethanol sensing was investigated [95]. The sensor was found to be sensitive to ethanol concentration in the range of 0–5 mM with a maximum sensitivity of 21.70 nm/mM. It was observed that the co-enzyme used in this configuration increased the sensitivity by almost 33%.

Muri et al. demonstrated a low-finesse Fabry–Perot cavity using a hydrogel half-sphere mounted on the fibre end face also coated with gold nanoparticles (GNP) and, therefore, combined interferometry and LSPR [72]. The free spectral range (FSR) and LSPR, resulting from the degree of swelling of the hydrogel while increasing the ethanol concentration from 30% to 50%, were measured. This paper was previously discussed in Section 2.2. of this article on interferometric sensors. 

**Figure 14:**
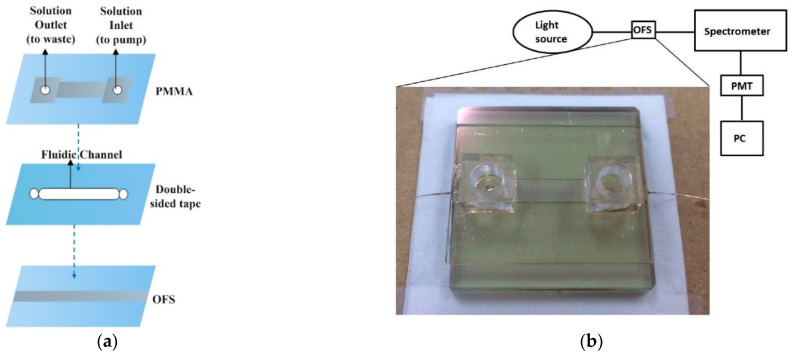

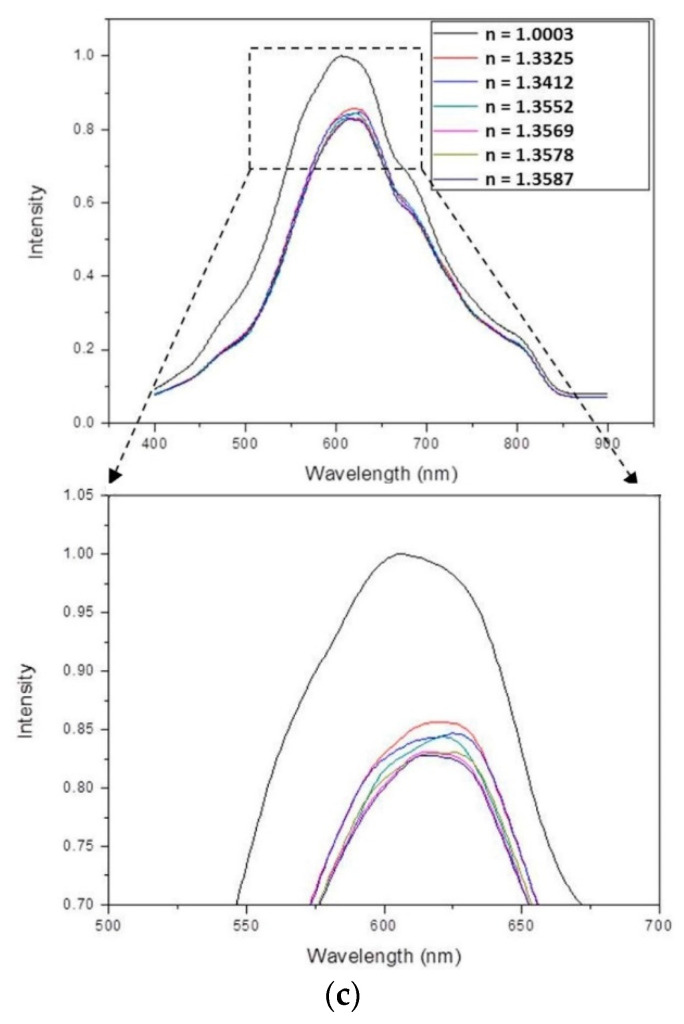
(**a**) Schematic representation of the microfluidic chip structure. (**b**) Experimental setup and photograph of the chip. (**c**) SPR resonance spectra for ethanol measurement. Reproduced from Ref. [38].

Sun et al. coupled a curved D-shaped optical fibre sensor with a microfluidic device for sensing bio-liquids with various RIs [38]. Multimode optical fibres were polished into a D-shape using the curvature trench method by embedding the fibre on a glass substrate. Then, a gold film of 10–12 nm was sputtered on the fibre-embedded glass substrate. A micro-fluidic chip was created using laser ablation to form patterns on a PMMA substrate and double-sided tape was used to secure the inlet and outlet ports of the fluid channel. The double-sided tape was subsequently used to bond to the D-shaped gold-coated optical fibre sensor for integration. The sensor was used to detect ethanol, methanol, ethanol–methanol and glucose solutions and the authors claim to have achieved a general maximum sensitivity of 10^−5^ RIU and 3.12 × 10^−5^ RIU specifically for ethanol. This corresponded to a minimum detectable limit of 0.06% (6 × 10^−4^) or 600 ppm ethanol. The integrated microfluidic chip achieved small size, low sample consumption and in-vivo measurement. The design of the microfluidic chip, experimental setup and SPR resonance spectra for ethanol measurement are shown in Figure 14 [38]. Jiang et al. proposed a novel U-bent POF LSPR sensor based on a graphene (G) and silver nanoparticles (AgNPs) structure [96]. A POF with 1 mm diameter was bent into a U-shape and a thin discontinuous layer of silver (Ag) was deposited on it. The thin layer of Ag was then coated with PVA (Polyvinyl alcohol)/G/AgNPs mixture by dip coating. They investigated different inner diameters of U-POF and thicknesses of Ag film for ethanol solution measurement in the range of 1.330 to 1.3657 RIU. The maximum sensitivity was measured as 700 nm/RIU. It was also observed that graphene improved the sensitivity of the LSPR sensor and delayed the oxidation process of the AgNPs [96], which causes aging of the sensor.

**Figure 15:**
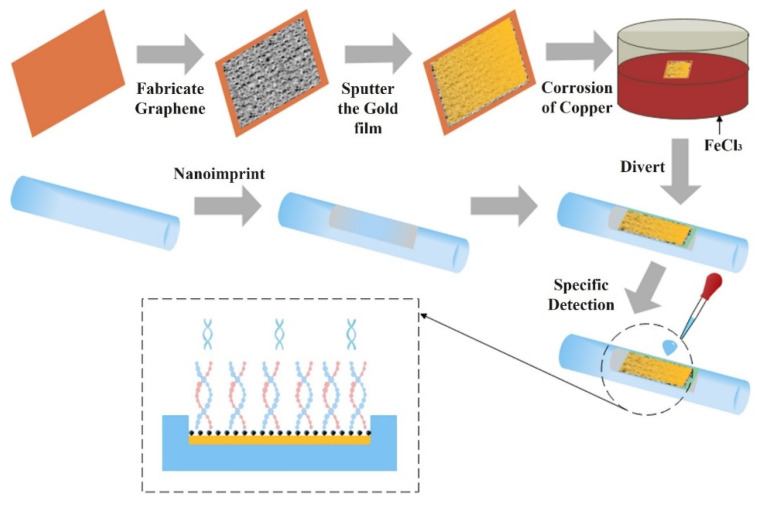
Preparation procedure of Au–graphene-based D-type fibre SPR sensor. Reproduced from Ref. [39].

**Figure 16:**
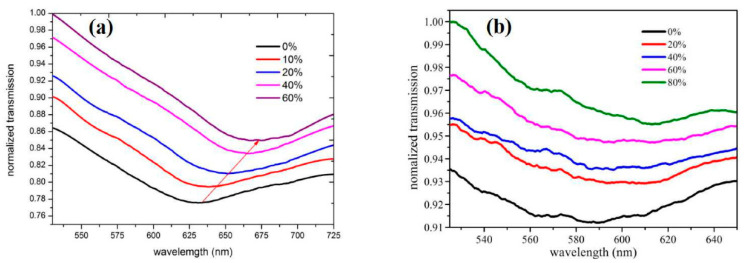
Transmission spectra of D-type fibre SPR sensor for different alcohol concentrations: (**a**) Au-graphene film-based, (**b**) gold film only. Reproduced from Ref. [39].

Sharma et al. investigated a samarium doped chalcogenide optical fibre used as an SPR sensor with a polymer cladding incorporating different coatings including an MoS_2_ base monolayer and a polythiophene (PT) layer coated over a 42-nm-thick Ag film for alcohols (ethanol + methanol) concentration measurement in water [97]. The sensor used an angular interrogation technique or selective ray (on-axis) method, which involved launching monochromatic light into the fibre core at different angles. This entailed mounting the laser diode light source on a rotary stage in order to launch the input light at different angles. The NIR signal was measured as a power loss (in dB) following transmission through the SPR interaction zone. The performance of the different configurations was compared quantitatively by formulating a figure of merit (FOM) for each one. The PT layer aided selectivity and allowed identification of the alcohols and provided an enhanced figure of merit (FOM). The authors claim significant improvement from previous work, i.e., 1647 FOM (RIU^−1^) at 1200 nm wavelength. It was also discovered that laser sources with longer NIR wavelengths resulted in an improved FOM and detection limit for the sensor. However, the sensitivity at longer wavelengths was lower, although this was compensated by a considerably higher accuracy. Xi et al. utilised a D-shaped fibre SPR sensor coated with an Au nanofilm–graphene layer [39]. The intended use was to detect highly sensitive DNA hybridization, but they were able to assess the SPR performance of the sensor using ethanol solutions with RI values ranging from 1.3330 to 1.3657 RIU. The graphene was grown directly onto copper and then gold film was sputtered on it, which ensured a better contact between the gold and graphene layer. The Au–graphene structure was finalised by etching the copper foil away using an FeCl_3_ solution. This process is shown schematically in Figure 15. Figure 16 shows the experimental results obtained by varying the ethanol concentration in the range of 0 to 80%, which gave rise to the RIU range specified above. The sensor produced a maximum red shift of 40 nm in the spectral dip minimum value over the range of concentration 0 to 80%. The sensitivity resulting from these measurements was determined as 1223 nm/RIU.

Guo et al. reported a novel lab-on-fibre approach for measuring RI to detect isopropyl alcohol, ethanol and methanol when they were applied separately on the sensor tip [98]. This lab-on-fibre sensor was designed by creating an array of nano cavities on the tip of a multimode optical fibre and then sputtering a 90-nm thin gold film on the side walls of the fibre and on the residual photoresist layer of 30 to 40 nm after etching process on the fibre tip, thus creating a conical cross-section of gold nanohole array. The etching process was implemented using electron beam lithography. In the case of ethanol detection, the sensitivity was measured to be 653 nm/RIU at a wavelength of 812.5 nm when the solution was changed from pure methanol to pure ethanol. The same measurements showed response and recovery times of 0.96 s and 4.1 s, respectively. It was demonstrated that both the conical cross-section of the gold nano-hole array and the underlying nano-cavities give rise to increased sensitivity.

It is clear that the SPR technique is capable of delivering accurate and highly sensitive optical fibre sensing solutions for ethanol concentration measurement. Recent advances in fabrication techniques have resulted in improved miniaturization which was exemplified in [98], where a lab-on-fibre ethanol sensor was reported. The combination of the progress in device and fabrication processes as well as significant improvements in computing capabilities for interpreting complex signals, e.g., spectral signatures using artificial intelligence techniques, means that there is significant potential for the incorporation of these techniques in future robust industrial measurement systems. However, some challenges remain particularly in achieving uniform and reliable thin metal coatings. The performance and various configurations of the Plasmonic Optical Fibre Ethanol sensors referenced above are further summarised in Table 5 in terms of sensor design, fibre type, metal coating, light sources and detectors, sensitivity (S), resolution (R), measurement range and type of application.

## 3. Current Status and Future Prospects of Optical Fibre Ethanol Sensors

The essential design parameters as well as the characteristics and the sensing performance of four major categories of optical fibre ethanol sensors, i.e., absorbance, interferometric, grating and plasmonic sensors, were described in detail and are summarised in Table 2, Table 3, Table 4 and Table 5. Clearly, a broad variety of optical fibre ethanol sensors, based on unique structural configurations and sensitive coatings, have been reported and their efficacy has been verified experimentally in the literature. Fluorescence-based optical fibre sensors have also been reported in limited cases, including [34,99,100]. Recent developments in whispering gallery mode type sensors have also been reported for ethanol measurement in aqueous solutions for which resonant modes are supported and the light signal circulates in a dielectric ring or microbubble. The supported modes form the ‘whispering gallery’ modes, leading to an enhanced light interaction length in the surrounding medium of the bubble or ring, which facilitates high-sensitivity ethanol concentration measurement [101,102,103,104]. Although the WGM sensors are a relatively recent development and have shown great promise in terms of enhanced sensitivity, their widespread use in industrial measurement is limited by the fact that the sharp (high Q) spectral resonance peak needs to be monitored using a high-resolution OSA or similar instrument. 

The diversity of sensor designs and configurations of optical fibre ethanol sensors is summarised in Table 6 and includes a summary of the advantages and disadvantages of the four main sensor categories considered in this review.

It is clear that optical fibre ethanol sensors have undergone remarkable development in recent decades in terms of sensing performance, robust design and miniaturization. A number of optical fibre sensors have shown great potential for ethanol concentration measurement in a wide range of real-world applications. This is based primarily on their characteristics, including potential low fabrication cost, small size, design flexibility, immunity to external electromagnetic interference and capability for long-distance sensing (from the point of measurement). Current research is mainly focused on making compact and flexible sensor structures using the wide range of different designs and available fibres as well as continuously striving to improve sensing performance using various sensitive materials such as novel 2D materials, new metal nano-films and nanoparticles and deformation (e.g., etching or tapering) of the fibre. However, this can often make sensor processing and fabrication complex and, in some cases, compromises the mechanical strength required for many industrial applications. Maintaining the uniformity and thickness of metal films and other coatings requires accurate processing with excellent repeatability. The repeatability is particularly significant in cases where novel 2D materials are used in combination with metal coatings to improve sensitivity. The coating process is often necessarily complex to achieve better contact between the metal and a functionalised sensitive layer. Cross-sensitivity to temperature and other chemicals in solutions containing ethanol is also a matter of concern as it can affect the selectivity and accuracy. Based on current developments and the perceived requirements of industrial applications, particularly with the arrival of Industry 4.0 and its relation to the burgeoning developments in the context of IoT, the following have been identified as significant future directions for research in optical fibre-based ethanol concentration sensors:

1. Targeting specific applications is crucial in bringing the technology to a real-world implementation. Being a good target analyte, it is necessary to explore and realise the application-specific requirements where ethanol concentration measurement is an important parameter, e.g., in biofuel production and processing, general fuel monitoring, the food and beverage industry, the paints and varnishes industry, medical diagnostics, and in the cosmetics industry. A specific example is evident in the case of bio-ethanol production using biomasses, specifically algae. There are very stringent requirements for specific sensitivity and resolution attainment, temperature stability and selectivity for sensing purposes as well as the ability to operate in real-time, which are crucial to avoiding sample loss and long lead time offline analysis [41,62]. It is clear that optical fibre sensors have a significant role to play in providing solutions in this scenario.
2. In many cases of industrial production and/or processing, high-sensitivity/ultra-low-level ethanol sensing is a fundamental requirement. Hence, many researchers have focused on improving the sensitivity of optical fibre ethanol sensors by seeking improvements in materials, coatings as well as sensor structures. However, temperature cross-sensitivity and/or cross-sensitivity caused by other chemical species in the measurand solution are also significant issues. Some researchers have formulated techniques to mitigate temperature cross-sensitivity, including an inline C-shaped open-cavity FPI [68], a combination of FP cavity and Michelson interferometry [69], micro grooved FBG [42], a combination of FBG and LPG for simultaneous temperature and RI measurement [37] and etched FBGs [84]. Cross-sensitivity due to other chemicals can be minimised or avoided by improving the selectivity of sensors and some work has also been reported for improving selectivity by modifying POF with CNTs [56], by combining interferometric and LSPR techniques [72] and by using ethanol-selective enzymes [94]. However, these sensor systems exhibit other drawbacks such as the use of specialised essential non-commercial parts, complex interrogation methods, low mechanical strength, narrow measurement range and limited sample-by-sample measurement. It is important to state here that some of those drawbacks may be irrelevant for some applications, depending on their specific requirements. Therefore, future research is likely to be focused on achieving high sensitivity combined with high selectivity and temperature compensation of optical fibre ethanol sensors.
3. Reusability of optical fibre ethanol sensors, specifically when they are coated with novel 2D materials and/or precious metals, is becoming increasingly important. In the case of SPR chemical sensors, some reusability techniques are explored such as removing the immobilised histidine-tagged peptide (HP) layer using imidazole (IM) on Ni metal to regenerate the sensor surface [105], by cutting and polishing the sensor tip to regenerate the surface [106] and by exposing the sensor to ethanol for repeated cycles [107] to demonstrate the reproducibility of results.
4. Distributed optical fibre sensing techniques for industrial measurement have gained significant attention during the last 10 years, which has culminated in many commercial systems becoming available (e.g., Luna [108] and OZ Optics [109]). Distributed optical fibre sensors are currently being used in several industrial applications, e.g., in oil and gas exploration, and in large structure monitoring. This technology certainly has scope for future measurements covering large scale ethanol industrial production and other chemical processing applications [110]. However, interrogation of distributed optical fibre sensors is complex and the instrumentation is currently expensive. Consequently, their uses are currently confined to a few ‘high end’ applications, e.g., where very large numbers of sensing points are required and non-optical measurement is not feasible. Research in this direction in combination with real-time, robust, sensitive and selective optical fibre ethanol sensing can help realise the Industry 4.0 requirements of real-time sensing and data collection to develop a digital twin of the facilities to predict yield and future demands of ethanol in industrial production and processing.

**Figure 17:**
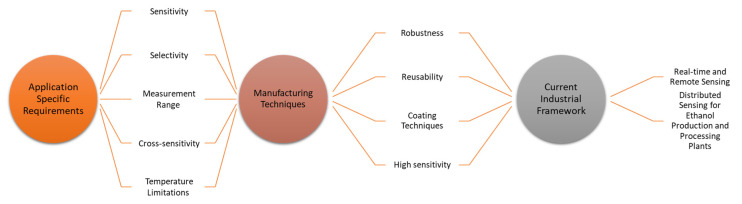
Interconnection of application-specific requirements, manufacturing techniques and the current industrial framework.

All these future directions and requirements of optical fibre ethanol sensors in the current industrial framework are interconnected. Knowing specific application needs can enhance the understanding of optical fibre ethanol sensor manufacturing techniques such as specific requirements for sensitivity, robustness, reusability, temperature limitations and coating materials and, hence, the industrial requirements for real time and remote sensing. Figure 17 explains the interconnection between the application requirements, sensor design, manufacturing techniques and the current industrial framework.

## 4. Conclusions

The four main optical fibre ethanol sensing techniques and their characteristics were discussed and summarised, i.e., absorbance, interferometric, fibre grating and plasmonic sensing, in terms of design parameters, fibre types, light sources and detectors, sensitive films/coatings, sensitivity, resolution, measurement ranges and applications. The advantages and disadvantages of all the types of optical fibre ethanol concentration sensors were also discussed with future potential research possibilities outlined. Enhancement of the sensitivity, selectivity, cross-sensitivity compensation and simplicity of manufacturing techniques can act as a driver for further improvement in optical fibre ethanol sensors, ensuring their advancement especially in the context of the specific modern-day industrial and technological needs.

Research progress shows that optical fibre ethanol sensors have the potential to be applied in broader industrially significant fields including biofuel production and processing, general fuel monitoring, food and beverages, paints and varnishes, medical diagnostics and cosmetics. High sensitivity, high selectivity and low cross-sensitivity are essential characteristics for implementation in real practical applications. In the future, the exploration of new structures and materials will intensify, but will remain subject to satisfaction of the above three performance indicators. Moreover, the current functionalization and biolayer deposition process of the majority of existing optical ethanol sensors are complex and the sensors are difficult to reuse. Therefore, the investigation of enhanced reusability may emerge as a significant research direction in the case of optical fibre ethanol sensors. Additionally, the growth in demand for multipoint and/or distributed sensing techniques for optical fibre ethanol sensors is likely to be a driver for the proliferation of these systems, particularly if the cost of interrogation is reduced through greater volume production and/or advancements in component technologies. The outlook for optical fibre-based ethanol sensing is, therefore, very strong in terms of both continued future research development as well as their likely penetration into growing industrial markets. 

## Figures and Tables

**Table 1:** Summary of commonly used ethanol measurement techniques.

Ethanol Measurement Techniques	Advantages	Disadvantages
Enzymatic method	Selectivity and sensitivity [6].	Low accuracy, reproducibility and enzyme stability issues. Non-specific interference [24].
Raman spectroscopy	Specificity and require small sample volume [19].	Precautionary measures required for laser use and difficulty to measure low concentrations of ethanol [18,19].
UV/NIR spectroscopy	Good sensitivity and less sample preparation. Non-destructive method [8].	Complicated calibration procedures, expensive and time consuming [20].
Dichromatic oxidation spectrophotometry	Inexpensive, high accuracy and do not require skilled analysts [25].	Sample loss and moderate time for analysis. Potassium dichromate oxidation: non-environmentally friendly due to the carcinogenicity of Chromium (Cr) (VI) [26].
Refractive index (RI) analysis	Simple and easy method [21].	Accuracy highly dependent on temperature and not suitable for complex solvent mixtures [21].
Gas chromatography (GC)	High accuracy and sensitivity [10].	Expensive instrumentation, laborious and long analysis time [27].
High performance liquid chromatography (HPLC)	High accuracy and reproducibility. Less time consuming in comparison to other chromatographic methods [22].	Expensive, requiring large quantities of expensive organics. Complex to troubleshoot problems [22].
Pycnometry	Simple method [12].	Long-time analysis, susceptible to error and requires experienced technicians and, hence, is expensive [12].
Densimetry	Rapid, accurate and simple method [12].	Requires large sample volume and pre-treatment process [20].
Hydrometry	Easy to use and inexpensive [12].	Requires large amounts of samples and is susceptible to user error [25].
Capillary electrophoresis	Inexpensive and quicker than HPLC [22].	Low reproducibility issues [28]. Lower accuracy than GC and HPLC [22].
Colorimetric methods	Requires small quantity of sample and is sensitive [29].	Non-selective and requires pre-distillation of sample [29].
Hydrogel-based and piezoresistive pressure sensors	Low cost, small size and inline process capability [23].	Measurement uncertainty [23].

**Table 2:** Summary of absorption-based optical fibre ethanol sensors.

Sensor Design	Fibre Type	Sensitive Coating	Light Source and Detector	S *	R **	Measurement Range	Application	Ref.
Tapered	Chalcogenide glass fibre (400 μm and 200 μm taper)	None	Infrared Light and Mercury–Cadmium Telluride Infrared Detector	-	-	5–50%	General	[57]
U-, Coil- and Meander-Shaped	Quartz/quartz fibre (600 μm core)	None	Deuterium Halogen Lamp, 650 nm LED, SR2000-TR Spectrometer and UV enhanced silicon Photodiode (UVS-025)	-	10^−5^ RIU	0–10%	Fuel cell concentration	[54,58]
Straight Grapefruit type (PCF)	PCF (10 μm core and 121 μm cladding)	None	Light Source of 632.8 nm	0.461 dB/vol%	-	0.1–1%	Biosensing	[59]
Tapered	Multimode (MM) Silica fibre (62.5 µm core and 125 µm cladding)	None, Graphene and GO	Tungsten Halogen Lamp and Ocean Optics USB4000 Spectrometer	0.829/vol% for graphene-coated sensor and 1.330/vol % for GO-coated sensor	-	5–40%	General	[35,60]
U-bend	MM PCS Fibre (62.5 µm core and 125 µm cladding)	GO	White LED and PG2000 Spectrometer	-	-	10–100%	General	[61]
Tapered U-bent	MM Silica fibre (62.5 µm core and 125 µm cladding)	MoS2	Broadband Light Source (450 to 1000 nm) Ideaoptics Instruments PG2000 Spectrometer	0.34 (∆A%/∆C%)	-	0–100%	Biosensing	[55]
U-bend	MM POF (980 µm core and 1000 um cladding)	None	Tungsten Halogen Lamp, 659 nm Photodiode and Ocean Optics QE65000 Spectrometer	817.760 O.D/RIU	10^−7^ RIU	0.005–0.05% (*w*/*w*) LOD: 9.2 × 10^−7^ RIU	Bioethanol production	[41,62]
Unclad Straight	MM POF (980 um core and 1000 um cladding)	Carbon Nanotubes (CNT)	Tungsten Halogen Lamp and Ocean Optics USB4000 Spectrophotometer	0.678/vol%/0.2%	-	20–100%	General	[56]
U-bent	MM PCS Fibre (62.5 um core and 125 um cladding)	Gold nanoparticles on Tungsten disulphide (AuNPs on WS2)	HL200 Light source (360 to 2500 nm) and Ideaoptics Instruments PG2000 Spectrometer	0.65 (∆A/∆C)	-	10–80%	General	[63]

* Sensitivity, ** resolution.

**Table 3:** Summary of interferometric optical fibre ethanol sensors.

Sensor Design	Fibre Type	Sensitive Coating	Light Source and Detector	S *	R **	Measurement Range	Application	Ref.
Single-arm common-path interferometer	PCF (LMA-10)	None	Power Meter	-	2.6 × 10^−5^ RIU	-	General	[67]
Step structure fibre inline MI	SMF-28 (core/cladding diameter of 8.2/125 µm)	None	Broadband Light Source and Spectrum Analyser (OSA, AQ6319)	-	-	0–50%	General	[71]
Inline C-shaped open-cavity FPI	Fused Silica tube for C-shaped cavity and SMF-28 (core/cladding diameter of 8.2/125 µm)	None	Broadband Super-Luminescent Diode SLD (1420 nm to 1620 nm) and Spectrum Analyser (OSA, AQ6370)	1368 nm/RIU	-	1.333–1.365 RI	General	[68]
Concave-core open-cavity FPI	PCF (38 µm solid core surrounded by 20 petals shaped airholes	None	Broadband Light Source (FiberLake-BBS) and Spectrum Analyser (OSA, AQ6370C)	1635.62 nm/RIU	-	0–19.11%	General	[36]
LSPR-based FPI	Double-Cladded Optical Fibre (DCOF) (DCF13, Thorlabs)	Gold Nanoparticles (GNP)	Light Sources (MBB1F1, 470–850 nm and S5FC1005S, 1550 nm Thorlabs) and Spectrometers (QE65Pro and NIRQuest-512-1.7 Ocean Optics)	-	-	30–50%	General	[72]
Singlemode–multimode–singlemode (SMS) MMI	SMF-28 and No-Core MMF (125 um diameter)	None	SLD (1465 to 1650 nm) and Optical Spectrum Analyser (OSA Anritsu MS9740A)	133.65 nm/RIU for 1.318 to 1.373 RI range and 390.88 nm/RIU for 1.373 to 1.420 RI range	-	1.318 to 1.373 RI, 1.373 to 1.420 RI and E50 to pure G87	Monitoring of gasoline/ethanol blends	[73]
Multimode–singlemode–multimode MI	SMF and MMF	Novolac Resin	1310 nm Light Source and Optical Power Meter	0.028972 dBm per % *v*/*v*	-	0–10%	Liquid phase alcohol detection	[74]
Taper-based MZI	SMF	None	-	28 nm/vol or 592.8 nm/RIU	-	30–70%	General	[43]
Michelson Interferometer (MI)	SMF	None	Broadband Light Source and Interrogator (1510−1590 nm)	885.437 to 1067.525 nm/RIU	-	1.3166–1.4346 RI	General	[69]

* Sensitivity, ** resolution.

**Table 4:** Summary of fibre grating optical fibre ethanol sensors.

Sensor Design	Fibre Type	Sensitive Coating	Light Sources and Detectors	S *	R **	Measurement Range	Application	Ref.
LPG	Bare LPG	None	-	-	-	0–100% ethanol in methanol	General	[77]
Etched FBG	Singlemode Ge-B co-doped photosensitive fibre (Newport F- SBG -15 and cladding diameter 125 ± 1 µm)	None	Broadband Light source and Optical Spectrum Analyser (OSA)	0.002 nm/%	-	0–50%	General	[78]
Microgrooved FBG	SMF (125 µm cladding diameter) with FBG of 22 µm period	None	FBG Interrogating System	-	-	Ethanol and 2.6% and 4.8% PVB in ethanol	General	[42]
LPG	SMF-28 with 21.6 mm long LPG of 540 µm period	None	Super-Luminescent LED and OSA	43 pm/%	-	20–40%	Monitoring of ethanol–gasoline blends	[79]
Encapsulated LPG	SMF-28 with 2.6 cm long LPG of 400 µm period	None	Broadband LED with centre wavelength of 1550 nm and OSA	Magnitude of 10 nm/RIU and 0.013 nm/%	-	Linear results in 0–70% for ethanol–water mixtures	Ethanol–water and gasoline	[80]
Etched FBG	Standard SMF based FBG1300 (Central Wavelength CW = 1308.49 nm) and FBG1500 (CW = 1539.87 nm) with pitch of 902.5 nm and 1062.5 nm, respectively	None	LED1 (Superlum, Pilot2, CW = 1544.2 nm), LED2 (Superlum, BroadLighter S-1300-G-I-20 SM) and OSA (Anritsu, MS9710B)	6.5 ± 0.2 nm/RIU (FBG1300) and 2.9 ± 0.2 nm/RIU (FBG1500)	-	0–100% for ethanol–water mixtures	General	[81]
Gold-coated FBG	Standard SMF and Commercial SMF FBG	Thin gold film	Halogen white light source (HL2000) and Ocean Optics Spectrophotometer (USB4000)	2% change in absorbance per 10% change in ethanol concentration ~0.2 (∆A/∆C)	-	0 to 99.7% ethanol in water	General	[82]
LPG and FBG	SMF 28	Cuprous oxide (Cu2O)	SLD, Broadband Source, OSA (AQ 6315B) and BraggMeter FS2200 SA	0.76 ± 0.01 nm/% *v*/*v* and 0.125 ± 0.003 dB/% *v*/*v*	-	1.5% *v*/*v* to 30% *v*/*v* ethanol in Gasoline	Quantification of ethanol–gasoline blends	[37]
Dual FBGs integrated in fibre ring laser structure	SMF 28 for FBG	None	Broadband light source and OSA (Advantest Q8384)	-	1.5 × 10^−4^ RIU	0–14% *v*/*v* ethanol in gasoline RON	General	[83]
Tilted FBG	FBGs and Tilted FBG with tilt angle of 6°	None	SLD, OSA and Photodetectors	-	1.5%	0–60% ethanol in gasoline	Gasoline quality monitoring	[84]
Etched FBG	Singlemode Ge–B co-doped photo-sensitive fibre (Fibre Core PS1250/150; cladding diameter ~125 µm)	Graphene oxide (GO)	Broadband ASE source and OSA (JDSU, MTS8000)	0.18 dB/percent	-	0–100% ethanol in petrol	Ethanol detection in petrol	[85]

* Sensitivity, ** resolution.

**Table 5:** Summary of plasmonic optical fibre ethanol sensors.

Sensor Design	Fibre Type	Metal Coating	Light Source and Detector	S *	R **	Measurement Range	Application	Ref.
Gold-coated unclad straight SPR sensor in a glass tube	NJ-PF200/300 (200 µm core diameter and 300 µm clad diameter)	Gold film	632.8 nm He-Ne Laser (Melles Griot V05LHR15), 50 cm focal length lens and ILX Lightwave (VOMM-6722B)	-	-	0–80% LOD: 0.5%	Ethanol content in liquor	[88]
Gold-coated cone-shaped SPR microdevice	Single-mode GeO2 doped silica core fibre	Gold film (13 nm)	Chopped Laser Source (780 nm) and Photodetector	-	10^−2^ RIU	-	General	[89]
Dual-colour SPR sensor	Step index multimode fibre (400 µm core diameter)	Silver and gold film (10 to 70 nm)	Tungsten halogen lamp, LEDs (612 nm and 680 nm) and Photodetector	-	-	0–50% LOD: 5.2 × 10^−4^ RIU	General	[90]
Conical shape SPR sensor	100 µm diameter optical fibre	Gold film (50 nm)	Semiconductor laser (690 nm wavelength) and PIN Photodiode	-	2 × 10^−4^ RIU	0.9 volume ratio of dimethyl sulfoxide and ethanol solution	General	[91]
Gold-coated straight SPR sensor fixed in a glass tube	NJ-PF200/300 (200 µm core diameter and 300 µm clad diameter)	Gold film (45 nm)	LEDs (563 nm, 660 nm and 940 nm) and Photodiode	-	10^−4^ RIU	0–50%	Ethanol content in spirits	[92]
Tapered fibre LSPR sensor	Single-mode optical fibre (SMF28e)	Star-shaped gold nano particles (80 to 120 nm)	Bromine tungsten light source (BFC-445), Monochromator (SBP500), Side window detector photomultiplier (PMTH-S1)	1190.5 nm/RIU	-	10–40%	General	[40]
Double-sided metal sputtered SPR sensor (inline transmission-based scheme and reflection-based scheme)	Polymer-clad-silica (PCS) multimode optical fibre (core diameter of 200 μm)	Thin gold film (50 nm), ADH and ADH/Nicotinic acid	Halogen lamp (Ocean Optics HL2000) and Spectrometer (Ocean Optics USB4000)	-	-	0–80%	General	[93]
Silver-coated SPR sensor combined with ADH and ADH/nicotinic acid enzymes	PCS fibre (core diameter of 600 μm)	Thin silver film (40 nm)	AvaLight-HAL tungsten halogen lamp, Microscope objective and UV-VIS-NIR Avaspec-3648 optical fibre spectrometer	-	-	0–10 mM	Ethanol in food and beverages	[94]
Silver/silicon/hydrogel layered SPR sensor with ADH and ADH/nicotinic acid enzymes	PCS fibre (core diameter of 600 μm)	Thin silver film (40 nm) and Silicon (8 nm)	Tungsten halogen lamp, Microscope objective and UV-VIS-NIR Avaspec-3648 optical fibre spectrometer	21.70 nM/mM	-	0–5 mM	General	[95]
FPI-based LSPR sensor	Double-Cladded Optical Fibre (DCOF) (DCF13, Thorlabs)	Gold Nanoparticles (GNP)	Light Sources (MBB1F1, 470–850 nm and S5FC1005S, 1550 nm Thorlabs) and Spectrometers (QE65Pro and NIRQuest-512-1.7 Ocean Optics)	-	-	30–50%	General	[72]
Curved D-type SPR sensor integrated with microfluidic chip	Multimode fibre (core diameter of 62.5 μm and cladding diameter of 125 μm)	Gold thin film	Tungsten halogen lamp (LS-1, Ocean Optics), Photoluminescence spectrometer (Triax 320) and Photomultiplier (R5108, Hamamatsu Photonics)3.	3.12 × 10^−5^ RIU ^1^	-	LOD: 0.06% or 600 ppm	General	[38]
U-bent LSPR sensor based on a graphene (G) and silver nanoparticles (AgNPs) structure	Plastic Optical Fibre (POF) with 1 mm diameter	PVA/G/AgNPs @ Ag thin film (3, 5, 6, 7 and 10 nm)	Light source (380 nm to 780 nm) and PG2000 spectrometer (Ideaoptics Instruments)	700 nm/RIU	-	1.330–1.3567	General	[96]
Samarium doped chalcogenide optical fibre SPR sensor (Ag/MoS2 monolayer/perfluorinated (PF) homopolymer layer/polythiophene (PT) layer) with angular interrogation technique	Samarium doped chalcogenide core/polymer clad	Ag (42 nm) MoS2 (0.71 nm) PT (~7 nm)	Laser diode and photodetector	177.18°/RIU (for ethanol in water) and 182.821°/RIU (for methanol in water)	-	Ethanol–water, methanol–water and ethanol–methanol binary mixtures LOD: 5.04 × 10^−6^ RIU (at ethanol in water) to 4.8 × 10^−6^ RIU (at methanol in water)	General	[97]
Au nanofilm–graphene D-type SPR sensor	POF (1 mm diameter)	Au and graphene	Light source (380–78 nm) and spectrometer (PG2000)	1223 nm/RIU	-	1.3330–1.3657 ethanol solutions	Specificity bioanalysis	[39]
Cavity-coupled conical cross-section gold nanohole array LSPR sensor	Multimode optical fibre (Corning Infinicor SX + 50/125) (core diameter of 50 μm and cladding diameter of 125 μm)	Photoresist (30–40 nm) Au (90 nm)	Broadband halogen light source and spectrometer (StellarNet, Inc.)	653 nm/RIU	-	-	RI sensing	[98]

* Sensitivity, ** resolution, ^1^ as defined by [38].

**Table 6:** Summary of advantages and disadvantages of four main categories of optical fibre ethanol sensors.

Sensor Type	Advantages	Disadvantages
Absorption-based sensors	Easy, simple, versatile and low-cost design. Ease of implementation. Reproducibility.	Fragility due to deformation of fibre. Low selectivity without a sensitive film.
Interferometric sensors	Robust and easily implemented. Multimode Interferometers: Easy design, flexible structure and reproducible. Sensitive.	Costly, precise and delicate design procedures for most interferometric techniques. Multimode Interferometers: non-periodic spectrum and, hence, difficult signal demodulation. Hydrogel-based FPI: difficult reproducibility.
Fibre grating sensors	Adjustable structure design. FBG and LPG: When combined, can be used for simultaneous temperature and RI measurement.	Require expensive interrogation systems. FBG: Fragile due to fibre etching for RI measurements and temperature crosstalk. LPG: Complicated signal demodulation.
Plasmonic sensors	Accuracy. High sensitivity.	High processing requirements in terms of uniformity and thickness consistency of metal coating.

## Data Availability

Not applicable.
